# Anticipating volcanic eruptions using rescaled range analysis of volcano-tectonic seismicity

**DOI:** 10.1038/s41598-025-28566-6

**Published:** 2025-12-29

**Authors:** Raúl Pérez-López, Carolina Guardiola-Albert, Alicia Felpeto, David Sanz-Mangas, Nahum Méndez-Chazarra, Rafael Abella, Miguel A. Rodríguez-Pascua, Julio López-Gutierrez

**Affiliations:** 1 Departamento de Riesgos Geológicos y Cambio Climático, Instituto Geológico y Minero de España – Consejo Superior de Investigaciones Científicas CSIC, Madrid, Spain; 2 Subdirección General de Vigilancia, Alerta y Estudios Geofísicos, Instituto Geográfico Nacional, Madrid, Spain; 3 Universitat de València, Valencia, Spain

**Keywords:** Rescaled range, Hurst exponent, Volcanic eruption, Earthquake, Forecasting, Canary Islands, Natural hazards, Solid Earth sciences

## Abstract

The possibility of forecasting volcanic eruptions remains a major challenge for the volcanological scientific community. To date, various techniques based on volcano-tectonic seismicity, endogenous gas emission and satellite imagery have been widely applied in an effort to understand and anticipate short-term volcanic behaviour leading to eruptions. The rescaled range analysis (R/S) applied to time series of volcano-tectonic earthquakes is a quantitative method for determining the short-term and long-term memory of seismic activity during volcanic unrest. By using the Hurst exponent, it is possible to identify the precise transition from anti-persistence to persistence in volcano-tectonic earthquake time-series (VT) associated with volcanic dike ascent. We calculated the Hurst exponent of volcano-tectonic earthquakes during the 2021 Tajogaite eruption (La Palma, Canary Islands), the temporal evolution of the GEOS diagram and its correlation with the sustained dynamics of the volcanic eruption. Our study suggests that the volcanic unrest system transitions from anti-persistence to persistence approximately two days before the eruption, indicating a non-return point and the imminent onset of the eruption. Furthermore, we identified five magma deep injections during the eruption. The final stage and potential cessation of the eruption can also be inferred from the asymptotic trend of the Hurst exponent.

**Supplementary Information:**

The online version contains supplementary material available at 10.1038/s41598-025-28566-6.

## Introduction

Forecasting volcanic eruptions is a critical area of research that seeks to mitigate the risks associated with volcanic hazards, which pose significant threats to human life and the environment. The need for accurate forecasting arises from the destructive potential of volcanic eruptions, including pyroclastic flows, ash fall, lava flows, and explosive eruptions, all of which can have devastating socio-economic impacts on nearby communities^1^. Accurate volcanic eruption forecasting remains challenging due to the complex and dynamic nature of volcanic systems, volcanic plumbing, magma unrest and uprising, variance in the stress field, unbalance threshold of magma buoyancy^2^. The variability in eruption precursors, both in terms of timing and intensity, makes it difficult to develop a one-size-fits-all prediction model^3,4^. The forecasting of volcanic eruptions involves the monitoring of various geophysical (i.e. surface deformation, earthquakes, etc.), geochemical (i.e. endogenous gas emission), and geological signals (i.e. environmental effects) that precede eruptions^5–8^. One of the most widely used methods is seismic monitoring, which detects the volcano-tectonic earthquakes (VT) caused by magma movement beneath the Earth’s surface. Swarms of volcanic tremors often indicate an impending eruption^9^.

In general, studies of pre-eruptive phases during volcanic crises related to VT earthquakes focus on establishing thresholds for daily earthquake counts, the shallowness of the focal depths, and seismic energy release, in coordination with GPS-measured surface deformation, diffuse gas emissions, and InSAR analyses^10–12^. Also, long-period earthquakes (LP) related to volcanic eruptions have been studied related with magma unrest^13–16^. Long-period (LP) and very-long-period (VLP) seismic signals are key indicators of volcanic activity, as they are typically associated with fluid movement, pressurization, and resonance within magma conduits or fractures, making them valuable precursors for impending eruptions. LP seismicity is widely regarded as a key indicator of fluid migration within the volcanic edifice, with source mechanisms typically attributed to fluid resonance in conduits and fractures^15,16^. Still, thresholds for early warnings in long-lasting eruptions have been detected by Langer et al.^17^. However, there is no model to determine thresholds for imminent volcanic eruptions over time, apart from probabilistic approaches based on empirical data and historical records. Even so, integrating tremor signals with SO₂ and radon emissions, as well as temperature anomalies, could help identify ‘failed eruptions’ based on their temporal patterns^18^. Identifying failed eruptions during periods of volcanic unrest is as crucial as forecasting and mitigating successful eruptions.

In this study, we applied Rescaled Range analysis (R/S)^19–22^ to the temporal distribution of VT earthquakes during the pre-eruptive phase of a volcanic unrest, to identify potential precursors indicating the likelihood of an imminent eruption. This analysis was combined with data on volcano-seismic energy release and the evolution of earthquake focal depths to characterize the different phases of a Strombolian eruption, and estimate the final phase of decreasing eruptive activity. The Hurst exponent, derived from R/S of time-series of VT earthquakes, measures the persistence or randomness of seismic activity over time. A significant change in the Hurst exponent, such as a shift toward more persistent (correlated) seismic patterns, can indicate increasing stress and magma movement within the volcanic system. By monitoring these shifts, we have identified precursory signals of an imminent eruption, allowing for timely warnings and mitigation efforts (between 48 h and a few days before the eruption). Additionally, by combining the Hurst exponent time variation with the b-value evolution in time (Maximum Likelihood Estimation, MLE) we can estimate the eruptive phases of magma feeding from the reservoir to the magmatic chamber, and the stress relaxation during the volcanic eruption.

## The 2021 tajogaite volcanic eruption

La Palma Island is one of the most active volcanic islands in the Canary Islands (Spain)^22–25^. In 2021, a magma chamber located at a depth of 9 kms exhibited signs of unrest over a few days, triggering a VEI 3 volcanic eruption in one-week later^26^. This volcanic eruption lasted for almost 85 days, forming a monogenetic volcano (cinder cone) with a height of c.a. 200 m and a basal crater diameter of 250 m, and with several vents trending NW–SE over a length of 650 m^27,28^. Additionally, a vast lava field covered approximately 1,200 ha on the west flank of the island, affected more than 2,900 houses and 350 ha of plantation, and moving more than 7,000 people. The eruption had a severe impact on small towns, including Todoque and the neighbourhood^27–30^.

The eruption was characterized by a rapid ascent of magma over a few days from a magmatic chamber located at 9–11 km depth, and generating an intermittent effusive lava flows accompanied by violent and explosive Strombolian activity^31,32^. Episodic phreatomagmatic pulses were also observed at the east flank of the volcano. The authorities’ main concern was the critical task of delineating the potential area of eruption onset. At this critical moment, the collected information included a swarm of VT earthquakes that began on September 11, with more than 200 VT earthquakes per day and an average depth of 9 km, and surface deformation compatible with a magma body emplacement^31,33^. This seismic swarm rapidly increased in energy, and over time, the hypocentres became shallower. On September 15, the focal depths of VT earthquakes began to migrate upward, approaching the surface (approximately 1 km depth). By September 19, a magnitude 3.8 earthquake occurred at shallow depth, widely felt by nearby residents and scientists. By that time, the Spanish National Geographic Institute had recorded and located 1,500 VT earthquakes^33,34^. This day, the eruption began.

**Fig. 1:**
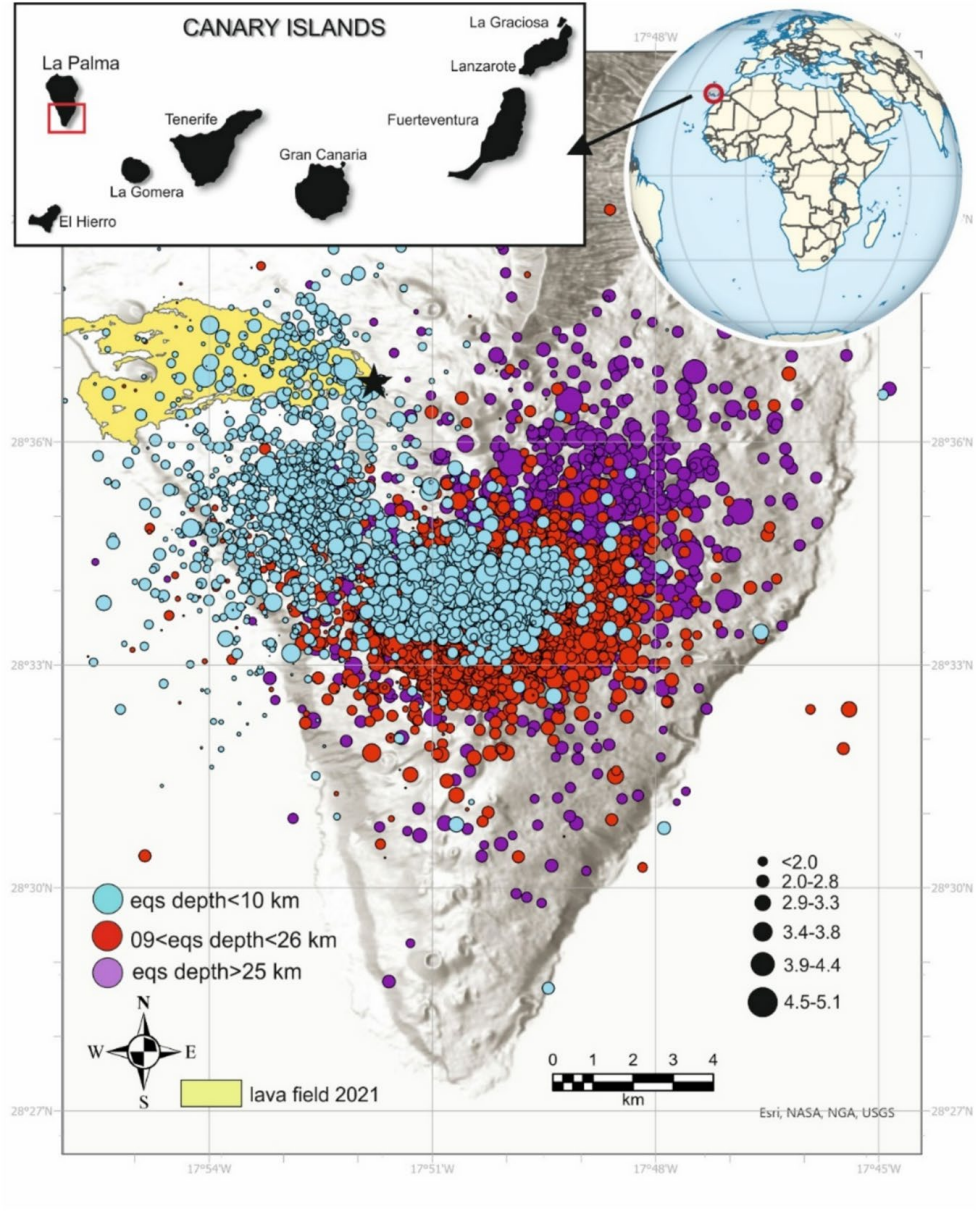
Spatial distribution of volcano-tectonic earthquakes (VT) on shaded relief recorded during the Tajogaite 2021 eruption^34^ (from 01/09/2021 to 02/01/2022). Light blue dots represent shallow VT earthquakes (d < 10 km), red dots denote earthquakes located between 10 and 25 km depth, and light purple dots indicate deep earthquakes with a depths greater than 25 km. The maximum magnitude of earthquakes recorded was m_bLg_ 5.1. The yellow area illustrates the lava field generated during the eruption, covering an area of 1,200 ha. The black star indicates the location of the Tajogaite cinder cone. The map was generated using ArcGIS Pro (Esri©), under a license provided by the Spanish National Research Council (CSIC).

**Fig. 2:**
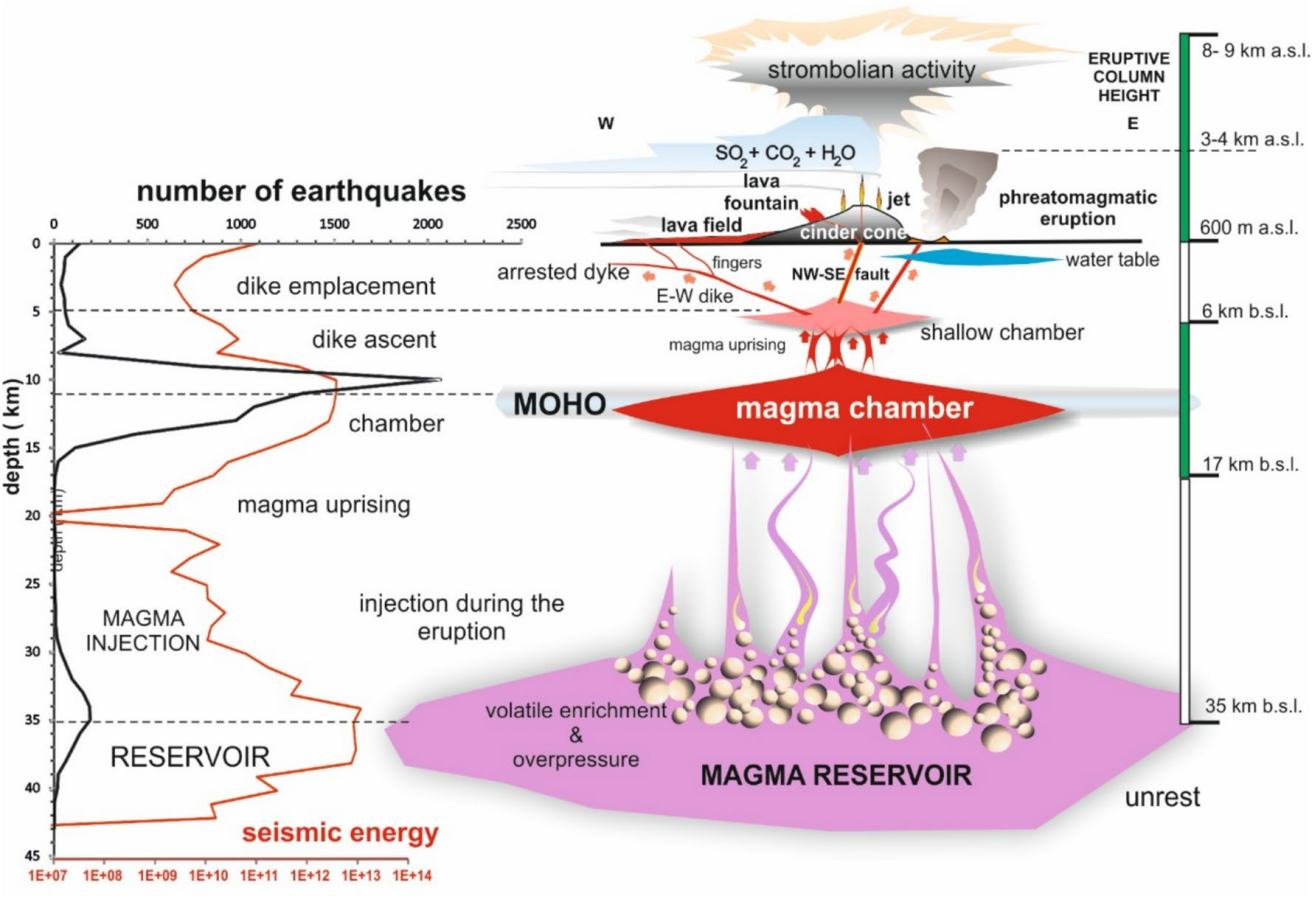
Left: Number of VT earthquakes by depth (km, black line) and seismic energy released during the volcanic eruption (red line). The magma injection originated from a large reservoir where primary melting occurs at depths greater than 25 km. The magma chamber is interpreted to be located between 9 and 11 km depth, near the Moho boundary, with a shallower chamber situated at approximately 5–6 km depth. The dike ascent to the surface occurred within a NW–SE fault^32^ that reached the surface eight days before the onset of unrest. Another E-W magma dike was emplaced during the middle of the eruption, with distal vents probably related to magma fingers^32^.

The spatial distribution of VT earthquakes during the eruption reveals magma movement from a magma chamber, located at a 9 km of depth, to the eruption site (indicated by the star in Fig. 1), along with a lava flow originating from a 600 m eruptive fissure flowing westward (Fig. 1 depicted by the yellow area). Subsequently, the 9 km-depth magma chamber was supplied by the magma reservoir at a depth of 25 km (Fig. 2, left curve). The magma ascent occurred through a NW–SE feeder dike, with several vents aligned NW from the cinder cone. Various magma injections led to a pulsating eruption, with a shift in volcanic activity from effusive to explosive, alternating lava fountaining with ash emission. Additionally, magma mineralogy was primitive, indicating a rapid magma ascent without assimilation and magma fractionation^35,36^.

The eruption of Tajogaite began on September 19, 2021, and concluded on December 13, 2021. During this period, approximately 9,105 VT earthquakes were recorded by IGN^34^, and categorized into three key depths (Fig. 2): (1) shallow earthquakes up to 9 km depth, (2) intermediate earthquakes between 10 and 25 km depth, and deep earthquakes with a depth greater than 25 km. More than 2,000 VT earthquakes were located between 9 and 10 km depth, with an accumulated seismic energy of 4.82 E + 12 J, and a total seismic energy of 6.68 E + 13 J (Fig. 2). The maximum magnitude recorded was m_bLg_ 5.1, with a magnitude of completeness (Mc) which oscillates between 1.9 and 2.7. Mc was calculated by plotting the frequency of the magnitude of earthquakes, cutting from the maximum frequency. The seismic network deployed by the Geographical Institute of Spain was improved during the volcanic eruption and, consequently, the Mc changed during the eruption time. The depth uncertainty of the hypocentres of VT earthquakes is not a single value, probably because they have used a different velocity model were used during the eruption crisis, as was pointed out by Del Fresno^33^. According to this data, the primary magma melting occurred at approximately 25 km depth, and the magma chamber was emplaced at approximately 9—11 km (Fig. 2) in coincidence with the Moho. The ascending magma from the chamber encountered a pre-existing NW–SE-trending shallow fracture^32^ or a low-density body, facilitating the rapid rise of a critical volume of magma to the surface and triggering the eruption^37^.

The eruption resulted in the formation of a monogenetic cinder cone of basaltic composition^27^, with a syn-eruptive stage characterized by multi-vent-opening^38^ and a post-eruptive stage that resulted in an ephemeral fumarolic activity (solfatara-type) and degassing^39^. The eruption dynamics shows three alternating eruption styles: (1) an effusive eruption characterized by high fluid lava emission (low-viscosity) and a maximum lava temperature of 1,090 °C (measured in the field by a thermocouple with a k-sensor). (2) Strombolian eruptions with a maximum column height of 9 km, large quantities of ash emission^40^, and metric-sized ballistic bombs ejected several hundred meters from the cinder cone. (3) Phreatomagmatic pulses on the east flank of the main vent possibly associated an underlying aquifer^41^.

During the pre-eruptive stage, the magma chamber experienced unrest on September 11, and the magma reached the surface eight days later after a shallow M 3.8 earthquake. The magma surfaced through a 4 km-long NW–SE dike emplacement. Eventually, a E-W dike later was emplaced with a lateral magma movement towards the west from the cinder cone, occurred from the last days of November, leading to the opening of several distal vents with low-viscosity and hot basaltic *pahoehoe* lava flows. Once the eruption began, episodic injections of deep magma reactivated and sustained its vigor and intensity for over 85 days.

## Results

In this section, we present and describe the main results obtained from the rescaled range analysis (R/S) and the temporal variation of the Hurst exponent calculated from the daily VT earthquakes. We also examine how these variations relate to the dynamic processes during the pre-eruptive, eruptive, and post-eruptive stages of the 2021 Tajogaite eruption.

### R/S analysis and GEOS diagram of VT earthquakes

The temporal variation of the Hurst exponent (H-exponent) can be represented by the GEOS diagram (Generalized Exponential Ornstein–Uhlenbeck Self-similarity)^19–22^. The GEOS diagram, introduced by Poveda and Mesa^22^, is defined as the ratio of the rescaled seismic energy range to the standard deviation, with the analysis time raised to the power of 0.5, R/S(t)/t^0.5^ (see Methods). This exponent generally trends toward a constant value, typically greater than 0.5 and often close to 0.75^42^. Temporal oscillations of the H-exponent reflect changes in the volcanic system dynamics. Assuming that VT earthquakes are closely linked to magma movement at depth, the H-exponent varies with the different phases of volcanic activity. Persistence in VT earthquake dynamics may indicate transitions in the volcanic system during an eruption. Accordingly, we use the GEOS diagram derived from VT earthquake data to characterize distinct eruptive phases, corresponding to specific volcanic events.

**Fig. 3:**
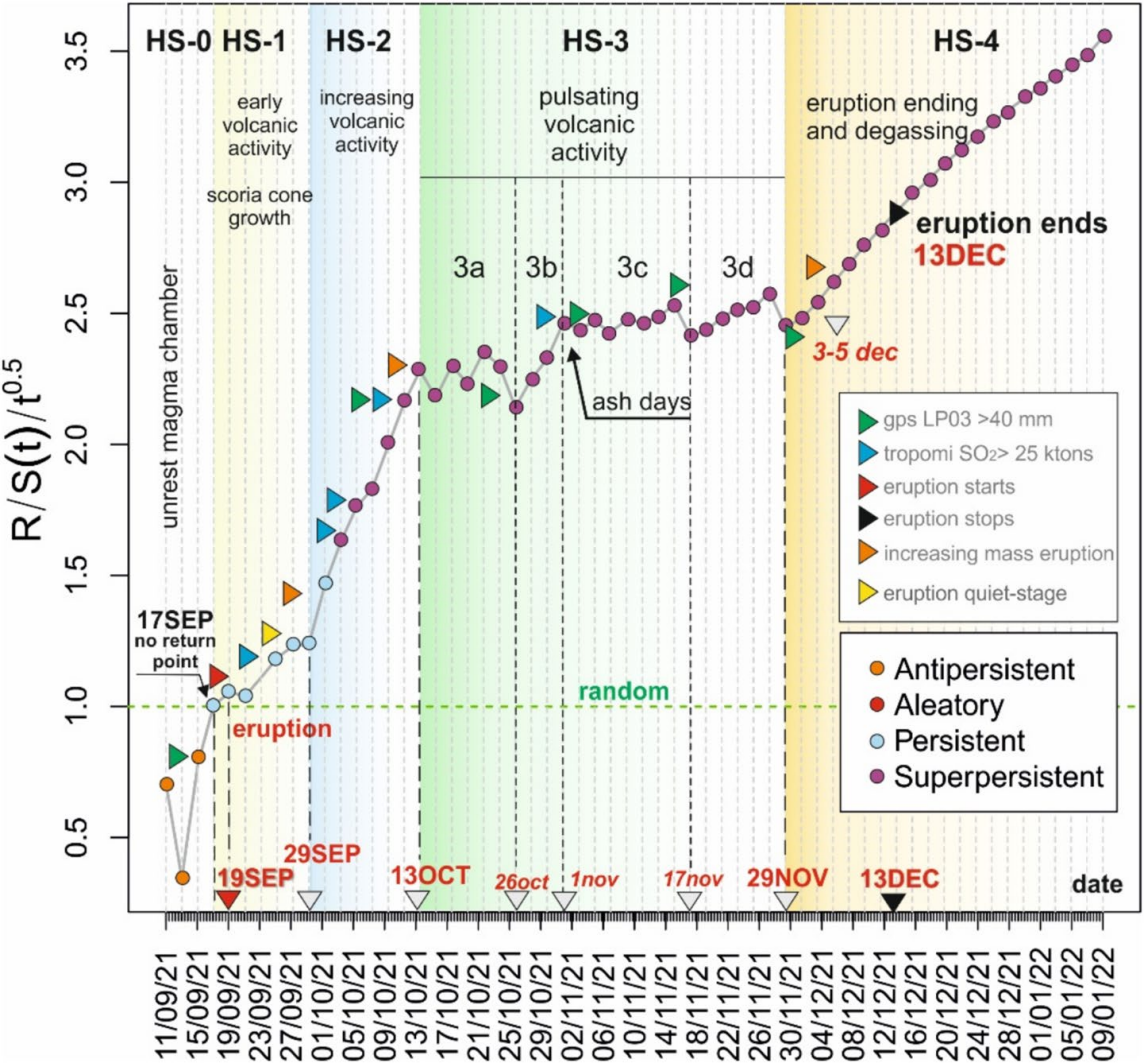
GEOS diagram illustrating the values of R/S(t)/t^0.5^ over time (t). Each data point represents a 48-h recording period of VT earthquake activity. The colour of each dot signifies whether the time series exhibits persistency or anti-persistency. Additionally, the randomness value is noted when R/S(t)/t^0.5^ equals 1, and when it surpasses 1.5, the behaviour is categorized as super-persistency. The volcanic main events presented in the figure were documented during fieldwork conducted during the volcanic eruption. Significant events such as GPS deformation and tropospheric SO_2_ signals from TROPOMI are also included into the diagram. Different stages (HS) have been identified according to the curve and main volcanic events (see text for further explanations).

We have developed an R code^43^ implementing an algorithm to calculate the GEOS diagram, the POX diagram, and the Mandelbrot and Wallis approximation of the Hurst exponent over time. In addition, the code plots the cumulative seismic energy (Benioff curve), computes the magnitude of completeness and the b-value from the seismic record, and determines the time required to reach the effective seismic range R(t) defined for each time step t. Applying this R-code^43^, we obtained the GEOS diagram of the VT earthquakes during the pre-eruptive stage (HS-0), the syn-eruptive stages (HS-1 to HS-4), and the post-eruptive stage (Fig. 3). We analyse the persistency and anti-persistency of the earthquake time series from the R/S analysis, defined by the value of R/S(t)/t^0.5^, anti-persistency when R/S(t)/t^0.5^ < 1 (orange dots in Fig. 3), randomness when R/S(t)/t^0.5^ = 1 (red dots in Fig. 3), persistency when 1 < R/S(t)/t^0.5^ < 1.5 (blue dots in Fig. 3), and super-persistency when R/S(t)/t^0.5^ > 1.5 (purple dots in Fig. 3). The algorithm we have implemented operates at a resolution of 48 h (two days). Working with logarithms and sexagesimal values in hours and minutes introduces complications that have so far prevented reducing the time window granularity to a single day. Regardless, 48 h before the eruption, we gain a new potential proxy for predicting incoming volcanic eruptions from a VT swarm.

We have divided the GEOS diagram according to the eruption stages observed in field work and sharp changes of the curve. Additionally, we have included relevant changes from the TROPOMI tropospheric SO_2_ signal (Supplementary Figure S1), and GPS daily variations (station LP03 GNSS^44^). Complete GEOS and Mandelbrot and Wallis (M&W) diagrams obtained each 7 days show the geodynamics of the eruption, long-term behaviour and singularities in the dynamic evolution of the eruption (Supplementary Figs. S2 and S3, respectively). The main parameters obtained for the complete time series of VT earthquakes during the Tajogaite eruption are presented in Table 1 (Supplementary Material).

**Table 1:**
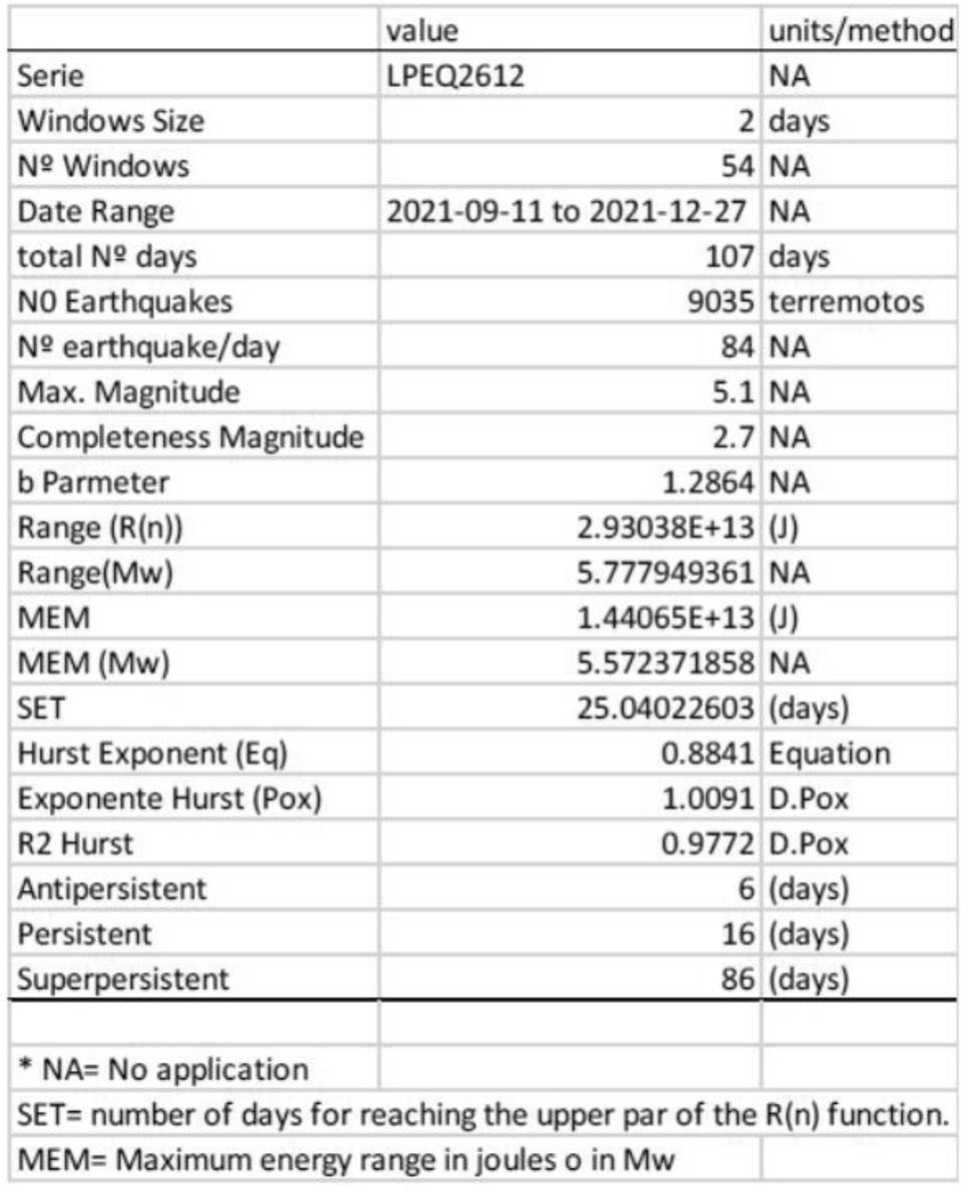
Main results from the R-code for the VT record during the Tajogaite eruption.

## Discussion

The GEOS diagram starts in an anti-persistent mode until September 17 (Fig. 3), when it reaches the persistency slightly above of the randomness position. From this point onward, the curve exhibits a general upward trend, with fluctuations marked by changes in slope and distinct peaks and valleys. Accordingly, we have divided the Hurst volcanic stages as follows:

Stage HS-0 (September 11–17), represents the pre-eruptive stage when the R/S(t)/t^0.5^ signal is less than 1. At this stage, the sequence is anti-persistent, indicating limited information about a potential eruption, and both the long- and short-term behaviour are unpredictable. The VT swarm maintains this anti-persistency dynamic until September 17 when the time-series crosses R/S(t)/t^0.5^ = 1, and the swarm shows a slight persistency. We interpret this moment as the "no-return" stage in the swarm, serving as a potential proxy for an imminent volcanic eruption. This proxy is supported by the shallowing of hypocentral depths, surface deformation (up to 12 cm uplift on September 17, at GNSS station LP03^44^).

Stage HS-1 (18–28 September), corresponds to the day of the volcanic eruption and the occurrence of a shallow m_bLg_ 3.8 earthquake, which was widely felt near the eventual eruption site. This stage is characterized by fast cinder cone growth, eruptive shockwaves, and the highest volatile pressure. On the day of the eruption (September 19), there is an initial decrease in the GEOS diagram followed by a rapid increase until September 27 when the eruption temporary paused (yellow triangle, Fig. 3). During this period, the TROPOMI data reveals an SO_2_ concentration surpassing 25 ktons per day (Supplementary data, Figure S1).

Stage HS-2 (September 29 to October 13), marked by an increase in the GEOS diagram until October 13, indicating an increase in eruptive mass and lava flows, while the TROPOMI data continue to register emissions above 25 ktons per day.

The subsequent stage HS-3 (October 14-November 29) is characterized by pulsating activity, which we associated with deep magma feeding linked to the occurrence of the largest magnitude deep earthquakes (Fig. 3). This stage displays a saw-shaped GEOS diagram, which we divided into four sub-stages based on to the curve shape, slope, and the GEOS R/S(t)/t^0.5^ values. These stages are HS-3a, HS-3b, HS-3c, and HS-3d. Stage HS-3a can be interpreted as pulsating activity recurring approximately every 48 h between October 13 and October 26. We infer a recurring 48-h deep magma feeding, manifested as earthquakes with m_bLg_ > 4.4 occurring an at depth around 25 km^34^. These earthquakes, reached a maximum magnitude of 4.8, occurred daily, and corresponded with peaks and valleys of the curve in the GEOS diagram.

Stage HS-3b is brief, similar in the eruption dynamics to stage HS-2, and is characterized by an increase in VT earthquakes and Strombolian activity accompanied by ash emission. During the stage HS-3c, VT earthquake activity intensified, with earthquakes reaching magnitude 5 and showing a higher daily R/S(t)/t^0.5^ values, coinciding with deep earthquakes of similar magnitude on November 29 (HS-3d). This period (HS-3c) marked the onset of the most significant seismic activity with magnitudes of 5 and 5.1, and depths exceeding 25 km. These events are interpreted as evidence of rapid magma-ascent to the main magma chamber, replenishing the system and sustaining the vigour of the eruption, characterized by alternating ash emissions and Strombolian explosive activity with several aligned jets, and reaching hundreds of meters in height. The stage HS-3c also shown sharp increase in GPS elevation data at the LP03 GNSS station^44^. The following stage HS-3d resembles HS-3c stage, featuring deep magnitude 5 earthquakes, but fewer number of events and with low magnitude in average. It ends on November 29 when deep seismicity ceases, and the overall seismic energy released diminished toward the end of stage HS-3.

Stage HS-4 (30 November- 13 December) represents the final stage, characterized by a decline in VT activity with earthquakes of magnitude < 4.5, a reduction in daily earthquake counts, a decrease in TROPOMI SO_2_ emissions (see Supplementary Figure S1), and no discernible increase in the GPS signal. In this final part of the GEOS diagram, sustained growth is observed with a super-persistent signal and consistent R/S(t)/t^0.5^ values. We interpret this continuous signal as indicative of the fading of volcanic activity and degassing, along with diminishing lava flow, reflecting the weakening of the eruption. Furthermore, the last violent eruptions on December 6 and 13 did not affect the GEOS diagram, suggesting they represent a singular and final episode rather than a signal of reactivation. We consider this event as a final violent pulse associated with the conclusion of the eruption. Notably, the slope of the curve gradually decreases to an asymptotic value in this stage. Poveda and Mesa^22^ interpreted this asymptotic GEOS behaviour as an indication that Hurst dynamics are no longer observed. This phase is characterized by residual earthquakes associated with final degassing and sluggish magma movement.

### Eruption Hurst stages and SSAM signal

Real-time Seismic Amplitude Measurement (RSAM) is a methodology widely employed during volcanic eruptions to monitor and assess seismic activity. It shows the overall amplitude of the seismic signal over time, highlighting changes in seismic activity. Seismic Spectral-Amplitude Measurement (SSAM), analyses the RSAM signal across different frequency bands, providing information on the spectral characteristics of the seismic signal. The RSAM method calculating the average seismic amplitude over a specific time window, typically one minute, expressed in terms of seismic energy. This measurement is valuable for tracking variations in volcanic tremor, which reflects changes in the magmatic system beneath the volcano. Endo and Murray^45^ and Chouet and Matoza^46^ expanded the application of RSAM for identifying changes in volcanic activity, including the onset of eruptions and the characterization of different eruption styles.

**Fig. 4:**
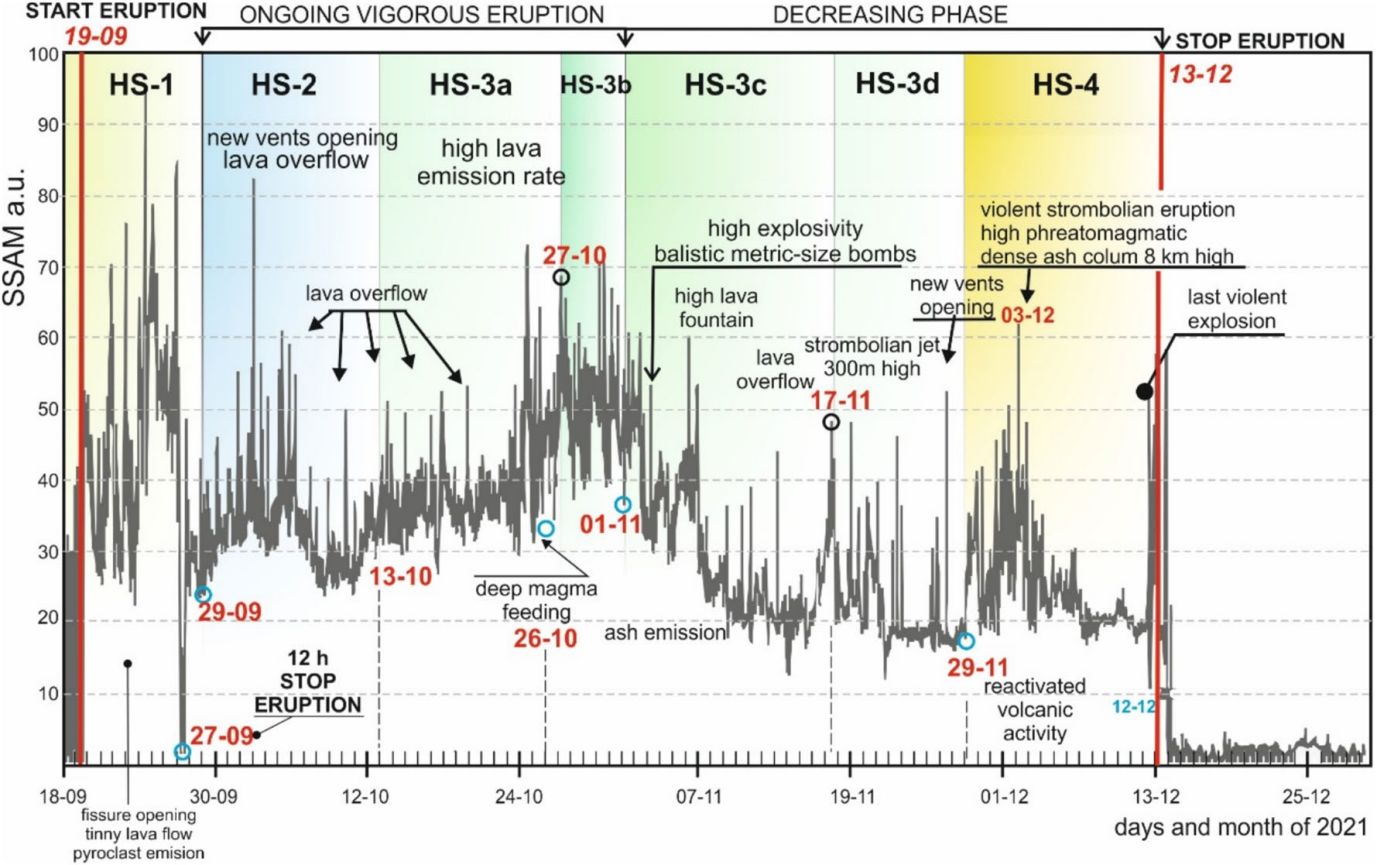
SSAM signal recorded by the Spanish National Geographic Institute (bold gray line), measured in arbitrary units (a.u.). Red vertical lines indicate the start date of the eruption (September 19) and the end of the SSAM signal (December 13–14). Blue circles mark dates of notable events during the eruption, changes in the lava-flow dynamics and high Strombolian activity. All eruption-related observations were made through fieldwork during the entire eruption. Numbers indicate the day and the month of 2021.

Figure 4 illustrates the evolution of the SSAM^47^ signal over time, along with the key volcanic events observed in the field during the Tajogaite eruption. The SSAM signal began on September 19, coinciding with the eruption onset, and concluded on December 13. The SSAM signal experienced a vigorous growth reaching its maximum value on September 25. The initial increase of the SSAM signal coincides with the anti-persistent part of the GEOS diagram, until September 17. Thereafter, the signal decreased until September 27 when the eruption briefly paused for a few hours with no observable volcanic activity. This oscillatory stage coincides with the stage HS-1 defined previously (see Figs. 3 and 4).

Eventually, the eruption vigorously resumed on October 29, with new vents opening along the NW dike emplacement, and followed by episodic pulses of lava overflows. SSAM signal peaks indicate lava overflows during HS-2 and HS-3, with an increase in lava discharge observed between October 25 and 31. During stage HS-3, deep magma input replenished the magmatic chamber. Volcanic activity declined after November 1 (HS-3c), although intense explosive pulses were recorded, with ballistic metric-sized bombs ejected several hundred meters from the cinder cone and vigorous lava fountains observed on the west flank.

Volcanic activity continued to decrease until November 15, punctuated by a sharp increase in VT earthquakes, associated with lava overflow, and probably linked to an increase in magma rate injection to the magmatic chamber. The formation of new vents related to an E-W dike emplacement from the main vent (cinder cone) characterize HS-3d. The final stage, HS-4, corresponds to a violent Strombolian eruption with phreatomagmatic pulses gradually waning until the early final days of the eruption, culminating with a violent explosion/paroxysm. Finally, the SSAM signal decreased to background noise from December 13 onwards.

### GEOS diagram and VT earthquake depth

The R/S analysis and GEOS diagram presented above were based on the complete VT earthquake database^34^ recorded between September 19, 2021 and January 9, 2022. However, as shown in Fig. 2, distinct depth concentrations can be observed in the volcanic swarm. The first depth range spans from 0 to 8 km, the second concentration occurs between 9 and 16 km, and the third one encompassed depths exceeding 25 km. These VT earthquakes depth clusters were also reported by Del Fresno^33^, who identified a shallow cluster at 11 km depth and a deeper cluster below 25 km. Additionally, during the eruption, a shallower cluster at 5–7 km depth remained active until September 27 (Fig. 2). Consequently, we filtered the VT database and organized according to these depth categories.

**Fig. 5:**
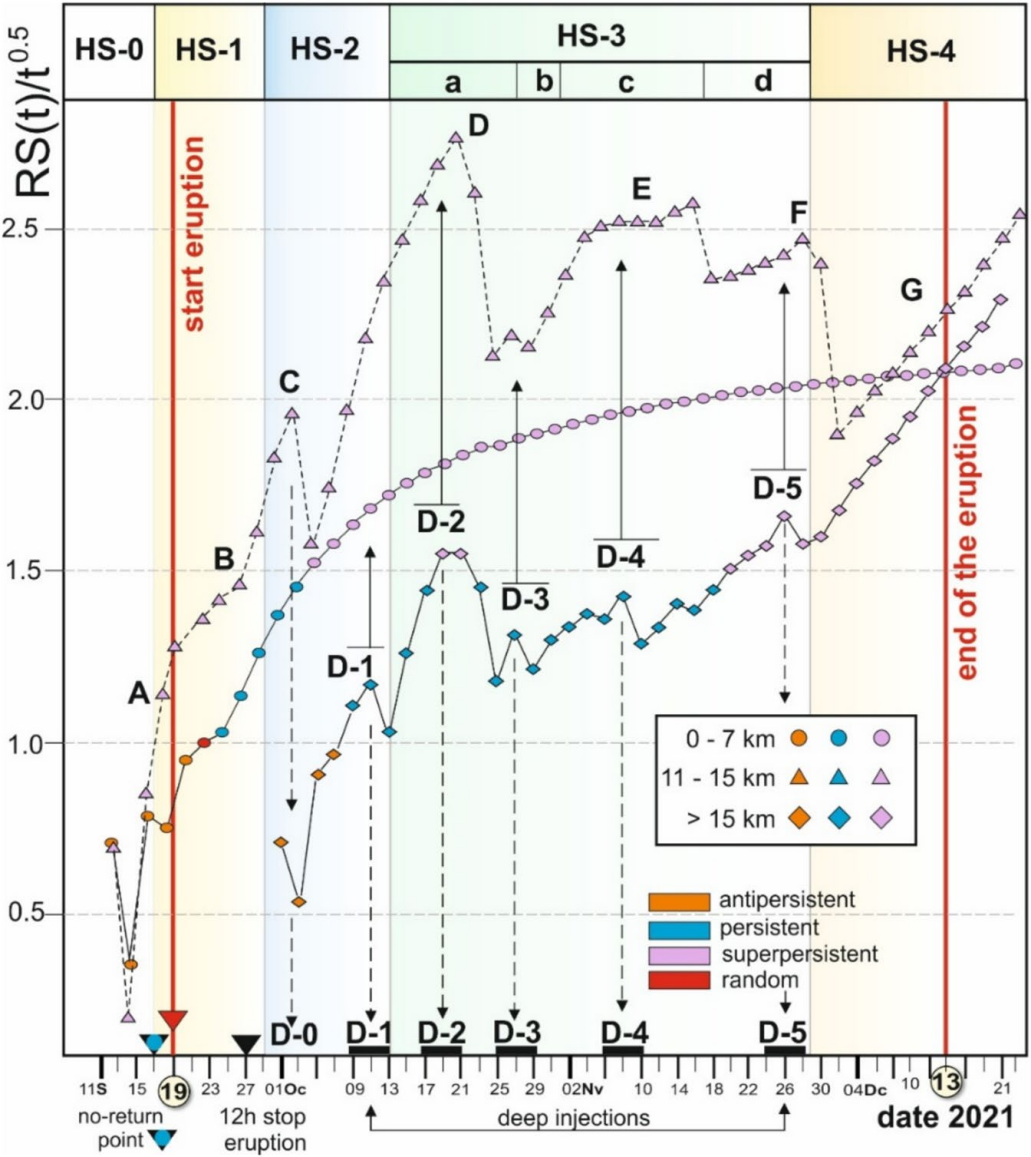
GEOS diagram for VT earthquakes filtered by depth. Circles represent shallow VT earthquakes related to the shallow chamber (depth 5–7 km) and dike emplacement. Triangles represent intermediate VT earthquakes (depth between 10 and 25 km), and diamonds represent the deep VT earthquakes (depth exceeding 25 km). The colour code is analogous to Fig. 4, and stages are differentiated accordingly. Lettered points signify specific dates, each of which is fully described in the text. D-# indicates dates of deep magma injections. D0 indicates the magmatic chamber pressurization.

By applying the R/S analysis and generating the GEOS diagram filtered by depth (Fig. 5), we can observe different curve behaviours that correlate with magma movement in the shallow chamber and dike emplacement for the first depth group (< 9 km), the main magma chamber for the second depth group (10—26 km), and the magma reservoir for depth exceeding 25 km.

Shallow VT earthquakes (circles in Fig. 5) associated with the shallow magma chamber ~ 5–7 km depth and the emplacement of NW–SE and E-W dikes, exhibit anti-persistent behaviour during the first ten days (from September 11 to 21), crossing the red line marking the eruption onset (September 19). The GEOS diagram shifts to a persistent pattern on September 23 (indicated by blue dots in Fig. 5), followed by gradual declines that correspond to the events of September 27, when the eruption paused. The curve’s persistency evolves into super-persistency from October 8 and eventually shows slight decrease, and settling into an asymptotic trend. We interpret this behaviour as the emplacement of a shallow magma chamber at 5—7 km depth, which emptied on September 27, causing the temporary cessation of the eruption. This shallow chamber was not refilled during the remainder of the eruptive period.

Intermediate depth earthquakes associated with the magma chamber at the Moho (at depths of 9—11 km, represented by triangles in Fig. 5) reached the "no-return" point (Point A in Fig. 5) 48 h before the eruption onset on September 19, followed by a decrease until September 27, when the eruption stopped for a few hours. From this date, the slope of the GEOS diagram increased, corresponding to the occurrence of deep VT earthquakes (Point B, GEOS diagram values represented by diamonds in Fig. 5). However, the dynamics of the deep VT earthquakes remained anti-persistent during the initial deep injection (D-1, Fig. 5), whereas intermediate depth VT earthquakes increased. In the GEO diagram (Fig. 5) shallow VT earthquakes showed an asymptotic trend until the end of the eruption.

Prior to this point, the VT signals from the magmatic chamber coincide with the onset of a substantial lava flow. This phase was accompanied by intense Strombolian activity, which continued until the shallow magma chamber depletion and the NW–SE-oriented dike was emplaced (Point C in Fig. 5). Thereafter, the deep injection replenished the magmatic chamber (stimulating primary melting), causing a sharp increase in GEOS diagram for intermediate-depth VT earthquakes. During this period (from October 1 to 3), new vents emerged along the NW–SE dike emplacement, likely linked to the influx of new deep magma into the dike and its lateral propagation.

The maximum GEOS diagram value for intermediate-depth VT earthquakes corresponds to the second-deep magma injection (points D and D-2, Fig. 5**,**). During this phase, the largest deep earthquakes occurred, accompanied with minor deep magma injections continuing until November 28 (D-3, 4, and 5, Fig. 5). Point D in Fig. 5 corresponds with the highly vigorous Strombolian stages and the largest primary melting recharge of the Moho chamber (9–11 km). Days of the eruption with high ash-plumes emissions correlate with the decline of the GEOS diagram between Points D and E.

Plateaus at E and F coincide with minor deep feeding, despite the occurrence of the largest magnitude VT earthquakes (m_bLg_ = 5 and 5.1) during this period (from November 3 to 30). The end of the F-plateau corresponds to the E-W dike propagation and the appearance of distal vents on the surface along this dike (magmatic fingers in Fig. 2).

Finally, the deep VT earthquakes exhibit regular behaviour from November 30, while the GEOS diagram for intermediate-depth VT earthquakes falls dramatically until December 2. Once again, the conclusion of the eruption displays no notable distinction in the regular increase of both GEOS diagrams.

The deep magma feeding inferred from the GEOS diagram (Figs. 3 and 5) coincides with the largest SO_2_ emissions (Supplementary Figure S1), exceeding 25 ktons/day. Specifically, the deep feeding episodes are as follows: D0 represents the initial magma chamber injection, on October 3 with 43 kTons/day of SO_2_ (see Figs. 5 and **S1**); D1 on October 7–9 with 32 kTons/day, D2 on October 19–22 (corresponding to point D in Fig. 5 with 24 kTons/day), D3 on October 25–26 (Fig. 5), with 15 ktons/day, D4 on November 4–6, with 28 ktons/day of SO₂, and finally, D5 on November 26–28, and 7 ktons/day.

We hypothesize that the shallow swarm (0–9 km) indicates the initiation of eruption dynamics through the rapid ascent of magma from the magma chamber (9–11 km depth). This initial magma volume was emplaced at a depth of approximately 5 km and subsequently ascended through a NW–SE-trending dike along a pre-existing discontinuity, likely a fault^32^. While the eruption could have concluded by October 1–3, the magma chamber was replenished by primitive, hot, and lower-viscous^36^ deep magma (primary melting or reservoir) in at least five pulsating episodes until November 30. Between each deep feeding episode, the volcanic activity alternated violent Strombolian stages with ash emissions and periods of effusive lava fountaining discharging from the west flank of the volcano, and flowing toward the west shoreline of the island. The advantage of the depth-filtered GEOS diagram lies in its ability to facilitate the interpretation of magma movement and plumbing from primary melting to the magma chamber, and ultimately to the surface, providing insights into the dynamics of the eruption evolution. The prolonged eruption, lasting 85 days, can be attributed to the deep magma injections, which extended the initial rapid magma ascent (until September 27 when the eruption paused). The evolution of the magma lava shown an early phase characterized by a lava relatively evolved in an amphibole-rich matrix until September 27^35^. Lately, eruption resumed, marked by an increase in maficity, interpreted by Ubide^35^ as magma recharge replacing the initial resident melt. We suggest that the shallow magma (5–9 km depth) experienced emptying and depressurization, followed by recharge from the initial pressurization at the Moho. Afterward, deep magma injections occurred, as suggested by Dayton^36^ based on clinopyroxene barometry analysis of lava and tephra. Finally, fractional crystallization of the magma chamber occurred between November 25 and 27, consistent with the timing proposed by Ubide^35^.

### Evolution of the Hurst exponent in time and depth, monitoring the eruption geodynamics

**Fig. 6:**
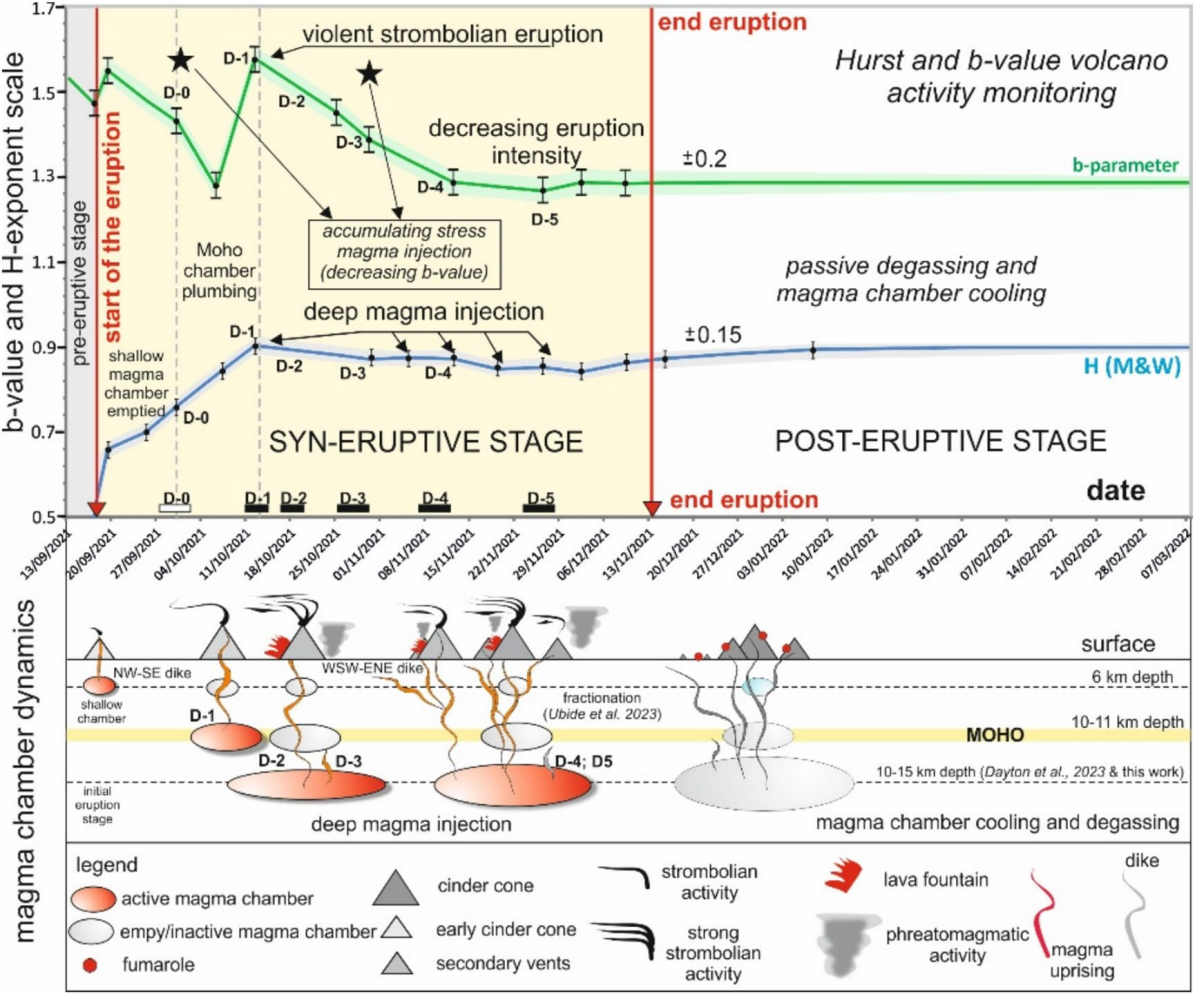
Time evolution of the Hurst exponent (H) from the Mandelbrot and Wallis diagram (M&W) (blue line), and the b-value from the VT seismic swarm (green line) during the Tajogaite eruption stages (pre-syn- and post-eruptive). We have included the eruption main events (starting, deep injection and eruption ending), according to the dates obtained from the GEOS diagram. The sizes of the ellipses representing magma chambers are not to scale. Vertical bars indicate the error in the b-value and H-exponent, and a shaded fringe has been included. Deep injections (D1 to D5) are included.

One of the advantages of the R-code (see Code availability) that we have designed is the ability to analyse the time variation of the Hurst exponent using the Mandelbrot and Wallis diagram (M&W), and the POX diagram^19–21^. We have used the M&W diagram for estimating the time-variation of the Hurst exponent, because its avoids non-stationary behaviour in earthquake time-series (see methods subsection). We obtained the M&W time evolution (Supplementary Figure S3) and compared it with temporal evolution of the b-value of the VT seismic swarm (Fig. 6). The resulting time evolution aligns with the H-exponent variation obtained from the M&W diagram. Furthermore, both curves correspond closely with abrupt changes in the b-value evolution of the entire VT seismic swarm over date.

The pre-eruptive stage is characterized by a b-value of 1.53 and a negative H-exponent (M&W), suggesting anti-persistence and an inability to gather information beyond the hypocentre location, released energy, and spatial distribution of VT earthquakes. The H-exponent (M&W), exceeding 0.5 (threshold for randomness), reached 0.6 on September 15 (Supplementary Figure S3), indicating long-term memory in the system. On the day of the eruption, the H-exponent increased to 0.66. We identify this stage as the onset of the eruption from a relatively evolved magma chamber.

Following this, the H-exponent (M&W) peaked at 0.9 in mid-October (12–15), coinciding with the onset of deep injections marked by violent Strombolian episodes. These injections are reflected in the curve by minor peaks associated with the five interpreted magma feeding injections. The decline in eruption intensity is indicated by a decrease of the b-value from 1.55 to 1.29, and a H-exponent (M&W) approaching to 0.9.

The end of the eruption is not indicated by a sudden shift in the H-exponent (M&W) or the b-value; rather, the curve becomes flat with no discernible variations (Fig. 6). However, it can be interpreted as a clear signal of the eruption’s conclusion, the depressurization of the magma chamber, and the initiation of the degassing and cooling stage (post-eruptive stage, Fig. 6). The time evolution of the H-exponent and b-value can be regarded as geodynamic monitoring, capturing the non-linear temporal behaviour of VT earthquakes during a volcanic crisis.

## How to use GEOS diagram in real-time

Using the VT (volcano-tectonic) earthquake records of a real-time seismic network, we first compile a dataset containing the following fields: date, time, latitude, longitude, depth (km), and magnitude. This dataset is then processed using the R-code provided by the authors, which generates the GEOS diagram by plotting one point for two days interval.

Initially, the GEOS diagram typically shows anti-persistent behaviour, indicating a self-regulating or oscillatory dynamic in VT seismicity. As time progresses, when the GEOS diagram reaches its first persistent stage (R/S(t)/t⁰·^5^ > 0.5), this point may be interpreted as a “no-return threshold”, signalling a high probability of an impending eruption, usually within 48 h to one week. If VT seismicity continues and the slope of the curve increases, the different slope phases may correspond to distinct volcanic processes, such as magma injections, fractional crystallization, or changes in eruption style (from effusive to explosive activity). The steeper the slope of the curve, the more intense and vigorous the eruption is expected to be. Finally, the convergence of the GEOS diagram toward an asymptotic value indicates the disappearance of the Hurst phenomenon^22^, which can be interpreted as the decline and eventual end of eruptive activity, with a residual level of VT activity that is clearly lower than the VT signal recorded throughout the entire eruptive and unrest phases.

Therefore, the GEOS diagram provides a novel means to anticipate both the onset of an imminent volcanic eruption during a shallow VT seismic crisis and the termination of eruptive activity, as indicated by its convergence toward an asymptotic trend.

## Conclusions

The eruption of Tajogaite at La Palma in 2021 was characterized by a rapid magma ascent, during which the Hurt exponent (H) of VT earthquakes firstly indicated anti-persistency dynamics within 6 days from the onset of magma chamber unrest. On September 17, six days after the unrest, the Hurts exponent increases to H = 0.6, suggesting a long-term memory in VT occurrence. The eruption began on September 19, with the opening of a vent along a NW–SE-trending dike. We speculate that September 17 is the no-return point, reflecting that the eruption was imminent (within a few days). The Hurt exponent continues to increase in time, reaching 0.86 on October 11–13, indicating super-persistence, and long-term memory in their dynamics. Eleven days after the onset of the eruption (September 30), deep magma injections from the large magma reservoir re-fed the magma chamber in at least five pulses, intensifying the violence of the Strombolian eruption and producing extensive basaltic lava-flows (1,200 ha) active for approximately 86 days. These five pulses persisted until November 30. Eventually, deep magma injection ceased, bringing the eruption to an end after three months of complex dynamics (Strombolian, effusive and phreatomagmatic). The decrease of the eruption intensity is also observed from the temporal variation H-exponent and the b-value, offering a proxy to the emergency authorities to estimate the potential end of the eruption. This proxy aligns well with INSAR and GPS data of surface deformation, SSAM signal and SO_2_ emission, showing robustness. Furthermore, the temporal variation of the Hurst exponent reflects the declining phase of the eruption, and help estimate the timing of its conclusion.

In conclusion, the rescaled range analysis (R/S) represents a significant advancement in volcanic eruption prevention and forecasting by providing a no-return date associated with an imminent volcanic eruption. When combined with other techniques such as surface INSAR and GPS monitoring deformation and measurements of diffuse endogenous gas emission (i.e. SO_2_ emission), the uncertainty in volcanic eruption prediction is reduced. This integrated approach enables critical decision-making by civil authorities and enhances crisis management by improving anticipation and situational awareness of impending eruption. When integrated with a real-time VT earthquake monitoring network at the volcano, this analysis yields a dynamic model for determining the current alert phase, providing valuable support for civil authorities in managing volcanic crises.

Moreover, this analysis reveals the geodynamic evolution of volcanic eruptions, highlighting relevant volcanic events related to deep magma feeding and the intensification of Strombolian/Hawaiian eruption stages in monogenetic volcanoes. It also provides insights into the duration of volcanic eruptions and the final post-eruptive phase, which corresponds to degassing and the cessation of volcanic activity.

## Methods

### Hurst exponent and GEOS diagram

The Rescaled Range Methodology, also known as R/S analysis, is a statistical technique used to study the long-term persistence or randomness in time series^19–21,42,48–51^. The primary goal of the methodology is to determine the underlying behaviour of a time series, especially concerning its tendency to exhibit long-term trends or mean-reverting behaviour. The key concept behind the Rescaled Range Methodology lies in the computation of the Hurst exponent, denoted as "H-exponent".

The Hurst exponent is a parameter that characterizes the fractal nature of a time series^19–21^. It provides insights into the self-similarity and long-range dependence properties of the data. The value of H ranges between 0 and 1. When H = 0.5, the time series behaves like a random walk, where future movements are entirely unpredictable, and there is no persistent behaviour. H values between 0.5 and 1 suggest that the time series has a long-term memory or exhibits persistence. A higher H value (closer to 1) indicates a stronger tendency for the series (super-persistency) to maintain its direction for an extended period, signifying a long-term trend. On the other hand, H values below 0.5 imply mean-reverting behaviour, where the series tends to reverse its direction after short-term fluctuations (anti-persistency).

The computation of the Hurst exponent^19^, involves the calculation of the rescaled range, which is a measure of variability in the data at different time scales. The method encompasses dividing the time series into multiple segments of varying lengths and computing the range (difference between maximum and minimum) for each segment. Then, the range is rescaled by dividing it by its standard deviation to reduce the influence of data magnitude. The rescaled range values are plotted against the segment lengths on a log–log scale, and the slope of the resulting line gives the Hurst exponent. The Hurst exponent has widespread applications in various fields: finance, hydrology, climatology, telecommunications, and more. It has been used to analyse stock market returns, river flow patterns, network traffic, and many other time series data to gain valuable insights into their underlying behaviour and predictability.

Let {X_k_} be a sequence of random variables, not necessarily independent, with some non-degenerate distribution. We define the n^th^ partial sum Y_n_ = (X_1_ + · · · + X_n_). Feller^51^ defines the adjusted range, R(n), as:

1 $$R\left(n\right)={max}_{1\le k\le n}\left\{{Y}_{k}-\frac{k}{n} {Y}_{n}\right\}-{min}_{1\le k\le n}\left\{{Y}_{k}-\frac{k}{n} {Y}_{n}\right\}$$

This variable R(n) was defined as “range” by Hurst^48–50^ and used the expression R(n) = max_1≤k≤n_{Y_k_} – min_1≤k≤n_{Y_k_}, and normalised the adjusted range by the sample standard deviation to obtain the Rescaled Adjusted Range statistic, R/S(n):

2 $$R/S\left(n\right)=\frac{R(n)}{\sqrt{\frac{1}{n}{\sum }_{k=1}^{h}{\left({X}_{k}-\frac{1}{n}{Y}_{n}\right)}^{2}}}$$

Hurst^48–50^ applied the R/S(n) in more than 600 natural time series of as river levels, rainfall, temperature, atmospheric pressure, tree rings, mud sediment thickness, and sunspots, and found a potential relationship between the variable “n” and the rescaled range, where:

3 $$R/S(n)\alpha n^{k}$$

The k exponent corresponds with the so called “Hurst exponent”. This is the mathematical basement for the Hurst exponent. Later, Mandelbrot and Wallis^19–21^ defined the M&W and POX diagrams to plot the Hurst exponent variation in time (see mathematical glossary). In the literature, there are different notations for the Rescaled Range: RS(n), R/S(n) and R*(n)^22,48–51^. We have used this formula for the algorithm in the R-code.

The GEOS (Generalized Exponential Ornstein–Uhlenbeck Self-similarity) and POX (Partition Function) diagrams are graphical representations used to analyse the long-term dependence of a time series and estimate the Hurst exponent^19^. The introduction of GEOS diagram^22^ show as a powerful tool the Hurst effect in non-stationary time series, to determine whether a given time series exhibit the Hurst effect, depending on the value of the scale of fluctuation^22^.

VT earthquakes time series data is stationary and free from any trends or seasonality^52^. We have obtained the b-value of the Gutenberg and Richter law^53^, the magnitude of completeness (Mc) and the study of the power-spectrum of the time distribution. The calculation of the GEOS and POX diagrams are divided in different steps: (a) Dividing the Time Series: we have split the stationary time series into different segments of varying lengths (scales). The lengths of the blocks should follow a geometric progression, such as powers of 2 (e.g., 2^0^, 2^1^, 2^2^, 2^3^, …), beginning for a time interval of two days. (b) Calculate the Range and Variance: For each segment, we have calculated the range (R) and the variance (V) of the data points within that block. (c) GEOS Diagram: by plotting the logarithm of the range, log (R), against the logarithm of the variance log (V) for each block on a scatter plot. The slope of the line that best fits the data points represents the Hurst exponent (H). (d) POX Diagram: Alternatively, we have obtained the POX diagram by plotting the logarithm of the variance log(V) against the logarithm of the block size log(N), where N is the number of data points in each block. The slope of the line that best fits the data points in the POX diagram also represents the Hurst exponent (H). Therefore, the Hurst exponent (H) can be estimated as the slope of the line in either the GEOS or POX diagrams. A slope of H = 0.5 corresponds to a random walk (Brownian motion), H < 0.5 indicates mean-reverting behaviour, and H > 0.5 suggests a long-term trend or persistent behaviour in the time series. It’s important to note that the estimation of the Hurst exponent using the GEOS and POX diagrams may be affected by the finite size of the data, noise, and other factors. Multiple data sets and statistical techniques are often used to enhance the reliability of the estimation.

The advantage of the GEOS diagram^22^ is that show in a simple plot the behaviour of the time-series around the randomness (R/S(t)/t^0.5 = 1), showing the anti-persistency when R/S(t) < 1, persistency when 1 < R/S(t) ≤ 1.5 and super-persistency when R/S(t) > 1.

Application of the R/S analysis and Hurst exponent in earthquake non-linear and statistical analysis can be consulted in^54^, where applied R/S analysis with a value of H ~ 0.72, greater than 0.5 and which they interpreted as earthquakes do not have a Poissonian memoryless distribution of time intervals; Hayakawa^55^ obtained the H-exponent for seismic swarms related to variations of the Earth magnetic field; Chen^56^ applied the R/S analysis in earthquakes and slip-predictable models; and Gkarlaouni^57^ for earthquake analysis related to the H-exponent variations in space and time in a wide area in Greece. All of these works investigate the long-term memory in the geophysical non-linear time-behaviour processes of earthquakes and volcanic occurrences. Regarding examples of the Hurst analysis applied in volcanology, there are the works of Morris^58^ about the roughness of lavas in Hawaii; Del Pin^59^ about the analysis to seismic data in a phase of possible unrest of the Teide – Pico Viejo volcanic complex; Olivé-Abelló^60^ applied the H-exponent of the time-series of thermohaline fluctuations in El Hierro; and Monterrubio-Velasco^61^ calculating the H-exponent and measuring the degree of persistence or randomness throughout an episode of volcanic emissions in Volcán de Colima.

We have used the R-code algorithm^43^ for computing the GEOS diagram with a time resolution of 48 h, to estimate the Hurst exponent from the POX diagram and the Mandelbrot and Wallis (1969) equation, the Range of the released seismic energy in joules, the b-value of the Gutenberg and Richter law^53^ (MLE), the number of earthquakes per day and the ordinary statistics of VT earthquakes (maximum value, mean and completeness magnitude). Also, the algorithm examines the power spectrum (β) of the time series to analyse the non-stationarity of the time series^22,52^.

Fractional Brownian Motion (fBm) is a process with memory, meaning past values influence future values, whose increments are Fractional Gaussian Noise (fGn), a self-similar, stationary Gaussian process characterized by the Hurst exponent (H)^62^. For fGn, the PSD (power spectral density) follows a power law: P(f) ~ 1/f^β^. White noise: has a constant power spectral density (i.e., β = 0). Pink noise (or 1/f noise): has a power spectral density that decreases with frequency, with −1 < β < 1 and β ≠ 0. Brown noise (or red noise): has a power spectral density that decreases even more sharply with frequency, with power proportional to β > 1. The β exponent of the time-series of VT earthquakes is −0.41 (Supplementary Figure S5), suggesting a pink noise for the VT earthquakes during the Tajogaite eruption.

In summary, the Hurst exponent is a measure of the temporal correlation or memory in a time series, and its relationship with fractal dimension depends on the nature of the fractal. Additionally, the Hurst exponent provides a way to categorize different types of noise, with white noise having H = 0.5, in a time series context, this implies that each data point is independent and uncorrelated with previous points. Pink noise having 0 < H < 1 (H ≠ 0.5), indicating a weak positive correlation between data points, and Brownian noise, also known as a random walk, having H = 1.0. It exhibits strong positive correlation, indicating a high level of persistence in the time series. These relationships provide insights into the structure and behaviour of time series data and are particularly relevant in fields such as geophysics, finance, and signal processing.

### Robustness of the R/S analysis and Hurst exponent

An extensive computer simulation carried out by Mandelbrot and Wallis^21^ shows the robustness of the analysis and the two warning cases, the Noah and Joseph effects. The authors shown that the obtained robustness of the R/S analysis extends to processes that are far from being Gaussian. They have represented the POX diagram for the time-series and the skewness and kurtosis values in white noise and random values, recognizing the so called Noah and Joseph effects. The Joseph effect is when the times series exhibiting noncyclic very global dependence. However, the Noah effect is referred to extremely non-Gaussian time-series with extreme events concentrated in non-cycling time intervals. This is the case of the VT earthquake swarms^63^.

### Non-stationarity of VT earthquake swarms

The main concern by applying the R/S analysis and calculate the H-exponent in earthquake swarms related to magma underneath movement is the non-stationarity, which means that statistical properties are changing through time. This could lead to produce unreliable and spurious results and leads to poor understanding and forecasting. Nevertheless, local stationarity involves assuming that the statistical properties of the time series are approximately constant within short intervals or local regions of the data. In other words, while the overall time series may be non-stationary, it is assumed to be stationary or nearly stationary within smaller segments. In the case of specific seismic swarms related to magma injection, the statistical properties are invariant in time, the b-value is robust, and the magnitude of completeness is invariable from a minimum number of earthquakes. Additionally, techniques such as rolling-window analysis, which involves updating the model parameters (magnitude and depth) at each time step using a fixed-size window of recent data, can help adapt to changes in local stationarity over time. Therefore, we have assumed a local stationarity for the seismic swarm related to the Tajogaite 2021 eruption. Graves^64^ have discussed the non-stationarity and stationarity of natural processes described as fractional Gaussian noise (fGn), for example, earthquakes, concluding that the physical explanation for non-stationarity and the assumption of long-term memory in Physics is still a matter of debate.

### Mathematical glossary

**R/S** = Rescaled Range analysis acronym.

**X(n)** = distribution of “n” variables in time. In this analysis, n = VT earthquake magnitude.

**R(n)** = range obtained as the difference between the Maximum and minimum value of a function of n variables in a time series Max{X(n)} – min {X(n)}.

**S(n)** = standard deviation of n.

**RS(n)** = **R/S(n)** = R*(n) = Rescaled Range or readjusted range by the expression R(n)/S(n).

**H** = Hurst exponent, defined as the slope of the fitted curve R/S(n) ~ n^H^.

**k** = ancient and original notation for the Hurst exponent (H).

**GEOS diagram** = a plot of R/S(n)/n^0.5^ *vs* n.

**POX diagram** = a plot of log(R/S(n)) *vs* log (n).

**M&W** = Mandelbrot and Wallis diagram of the H value in a time interval “τ”.

β= exponent of the power spectral density function (PDS).

## Supplementary Information

Supplementary Information 1.

Supplementary Information 2.

Supplementary Information 3.

Supplementary Information 4.

## Data Availability

The datasets of Volcano Tectonic earthquakes generated and/or analysed during the current study are available in the *Instituto Geografico Nacional*, repository, free available from IGN Catalog (https://www.ign.es/web/ign/portal/sis-catalogo-terremotos). Last access 2022a. 10.7419/162.03.2022

## Code availability

R-code for Hurst exponent for seismic time-series is available under reasonable request to the authors.

## Notes

The original online version of this Article was revised: The original version of this Article contained errors in the names of the authors Raúl Pérez-López, Carolina Guardiola-Albert, Alicia Felpeto, David Sanz-Mangas, Nahum Méndez-Chazarra, Rafael Abella, Miguel A. Rodríguez-Pascua, Julio López-Gutierrez which were incorrectly given as Pérez-López Raúl, Guardiola-Albert Carolina, Felpeto Alicia, Sanz-Mangas David, Méndez-Chazarra Nahum, Abella Rafael, Rodríguez-Pascua Miguel A. & López-Gutiérrez Julio.
