# Correction: Anticipating volcanic eruptions using rescaled range analysis of volcano-tectonic seismicity

**DOI:** 10.1038/s41598-026-43168-6

**Published:** 2026-03-09

**Authors:** Raúl Pérez-López, Carolina Guardiola-Albert, Alicia Felpeto, David Sanz-Mangas, Nahum Méndez-Chazarra, Rafael Abella, Miguel A. Rodríguez-Pascua, Julio López-Gutiérrez

**Affiliations:** 1 Departamento de Riesgos Geológicos y Cambio Climático, Instituto Geológico y Minero de España – Consejo Superior de Investigaciones Científicas CSIC, Madrid, Spain; 2 Subdirección General de Vigilancia, Alerta y Estudios Geofísicos, Instituto Geográfico Nacional, Madrid, Spain; 3 Universitat de València, Valencia, Spain

Correction to: *Scientific Reports* 10.1038/s41598-025-28566-6, published online 29 December 2025

The original version of this Article contained errors in the names of the authors Raúl Pérez-López, Carolina Guardiola-Albert, Alicia Felpeto, David Sanz-Mangas, Nahum Méndez-Chazarra, Rafael Abella, Miguel A. Rodríguez-Pascua, Julio López-Gutierrez which were incorrectly given as Pérez-López Raúl, Guardiola-Albert Carolina, Felpeto Alicia, Sanz-Mangas David, Méndez-Chazarra Nahum, Abella Rafael, Rodríguez-Pascua Miguel A. & López-Gutiérrez Julio.

The original Article has been corrected.

