# Supplementary material for: Anticipating volcanic eruptions using rescaled range analysis of volcano-tectonic seismicity
Source: Sci Rep. 2025 Dec 29;15:44803. doi: 10.1038/s41598-025-28566-6 (PMC12748848; doi:10.1038/s41598-025-28566-6)
Supplement: Supplementary file 1 — Supplementary Information 1. [file 41598_2025_28566_MOESM1_ESM.pdf]

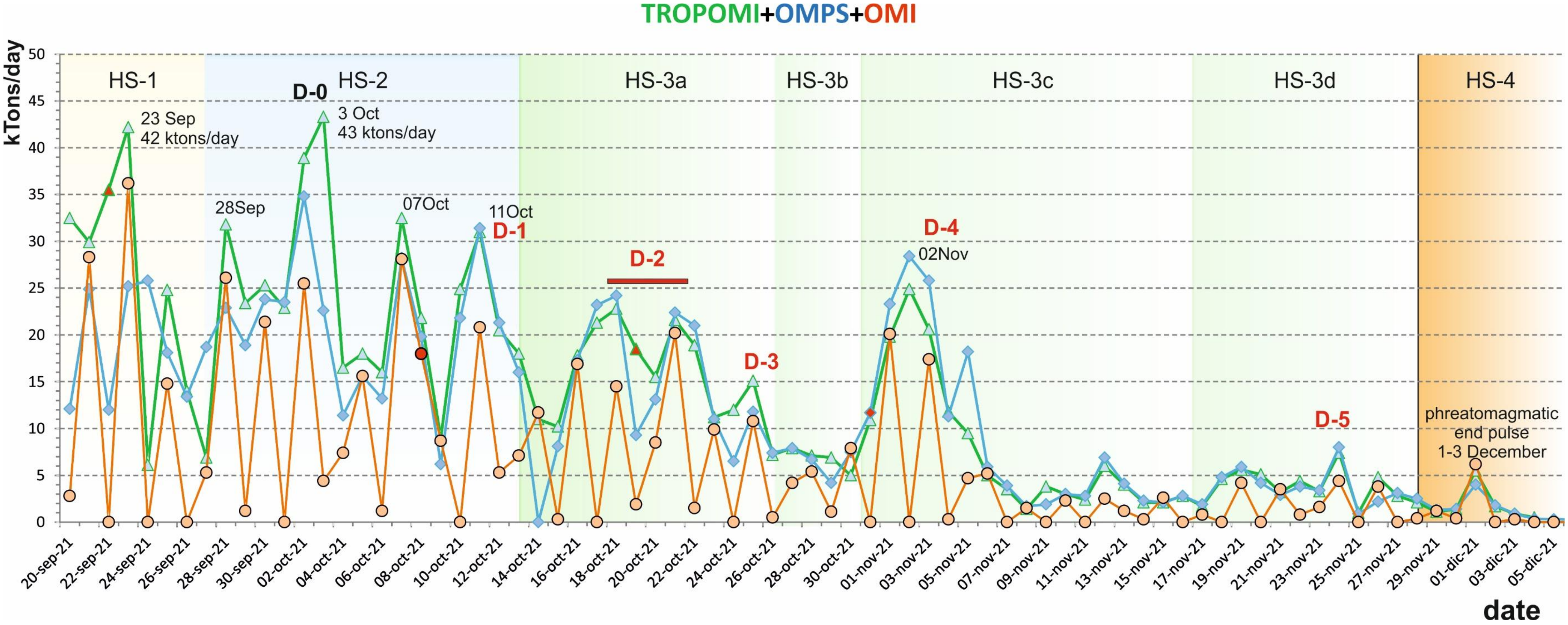


**Figure S1**. Tropospheric $SO_2$ emission during La Palma eruption (https://so2.gsfc.nasa.gov/pix/daily/1223/canary_1223z.html, last access January 2022). Green line corresponds to TROPOMI, blue line OMPS and red line OMI. HS stages have been obtained from SSAM (Fig. 3) and GEOS diagram (Fig. 4). Highest peaks of tropospheric $SO_2$ values (c.a. 43 ktons/day) appear in HS-1 and HS-2, in coincidence with the maximum slope of the GEOS diagram. $SO_2$ peaks on 07, 11 October, and 02 November correspond with deep magma injection and appear also in GEO diagram. DJ-# corresponds with Deep-Injection pulses described in Figure 5.
