# Supplementary material for: Anticipating volcanic eruptions using rescaled range analysis of volcano-tectonic seismicity
Source: Sci Rep. 2025 Dec 29;15:44803. doi: 10.1038/s41598-025-28566-6 (PMC12748848; doi:10.1038/s41598-025-28566-6)
Supplement: Supplementary file 2 — Supplementary Information 2. [file 41598_2025_28566_MOESM2_ESM.pdf]

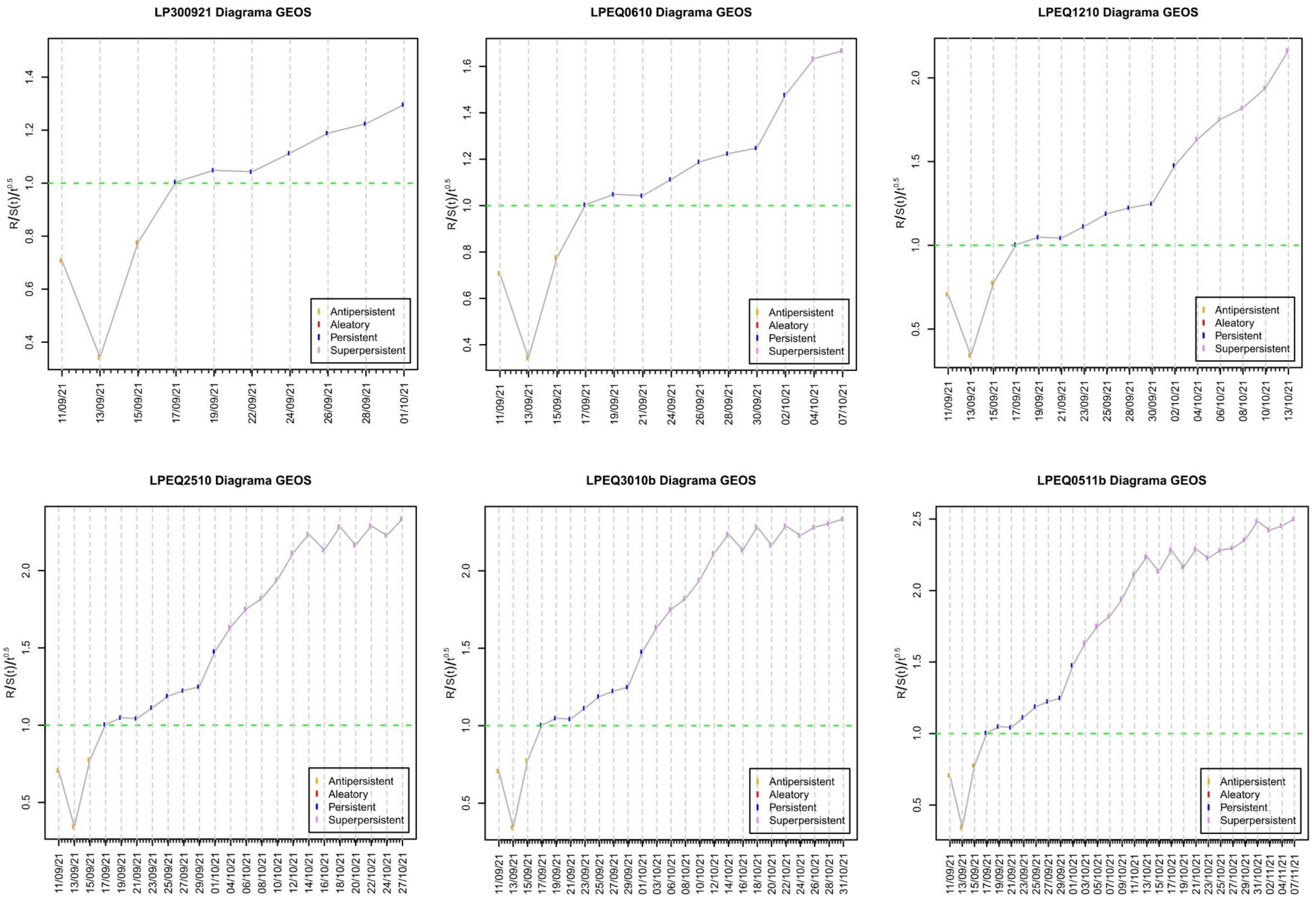
LP300921 Diagrama GEOS
LPEQ0610 Diagrama GEOS
LPEQ1210 Diagrama GEOS
LPEQ2510 Diagrama GEOS
LPEQ3010b Diagrama GEOS
LPEQ0511b Diagrama GEOS
R/S(t)/t^0.5
Antipersistent
Aleatory
Persistent
Superpersistent

**LPEQ1211 Diagrama GEOS**

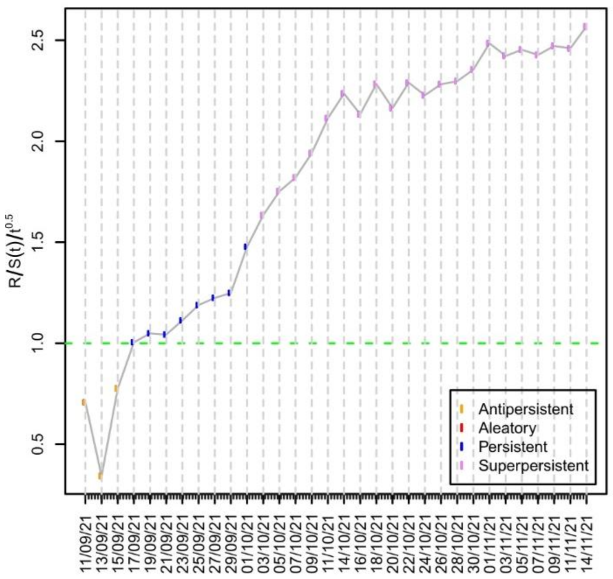


**LPEQ1911 Diagrama GEOS**

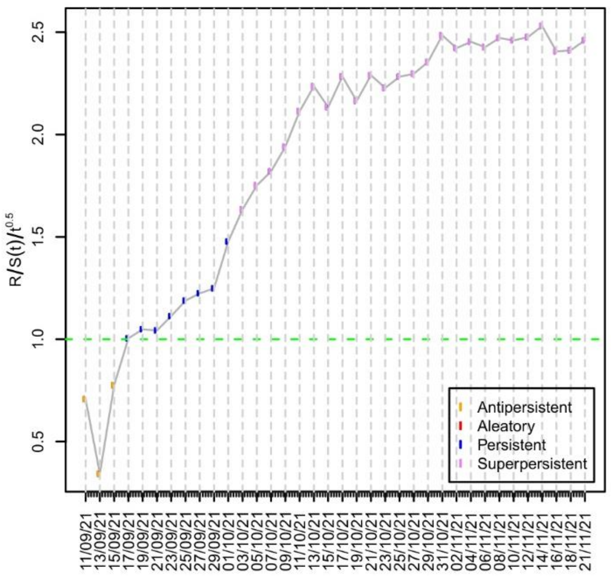


**LPEQ2611 Diagrama GEOS**

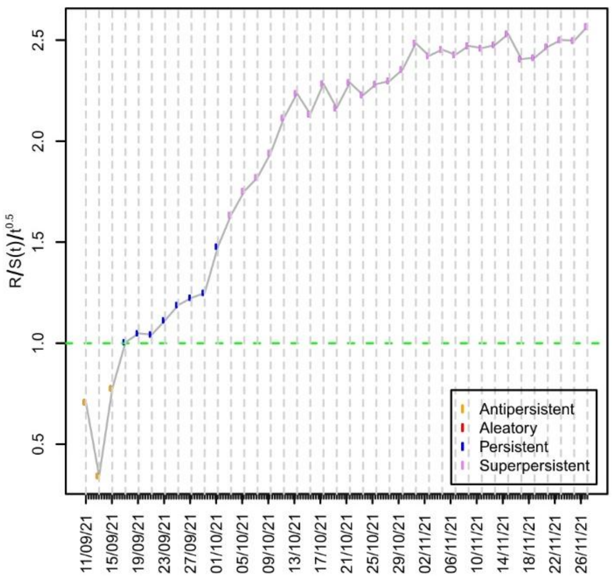


**LPEQ0212 Diagrama GEOS**

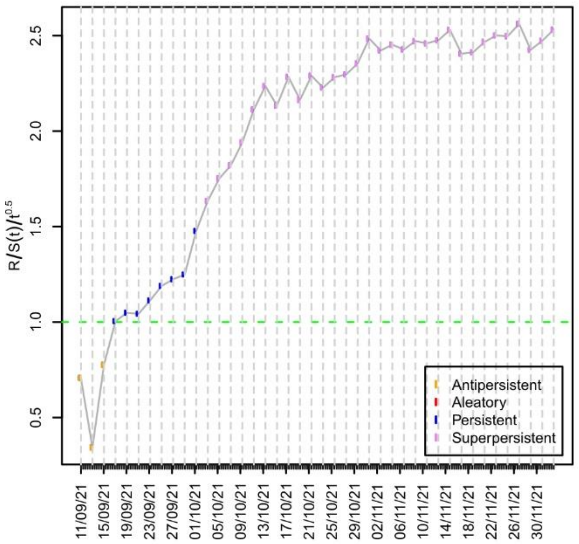


**LPEQ0912 Diagrama GEOS**

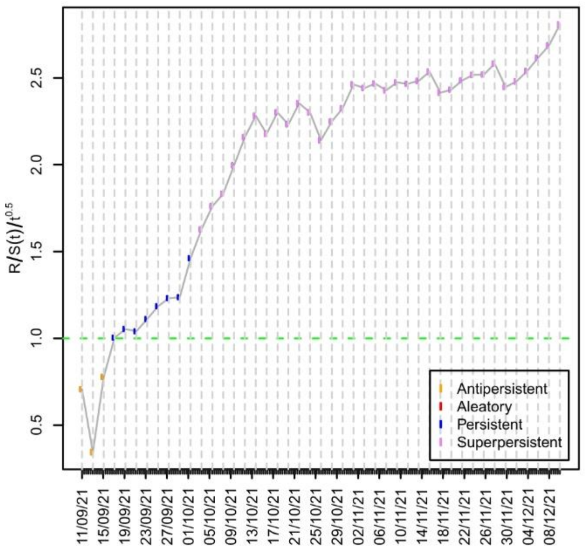


**LPEQ1512 Diagrama GEOS**

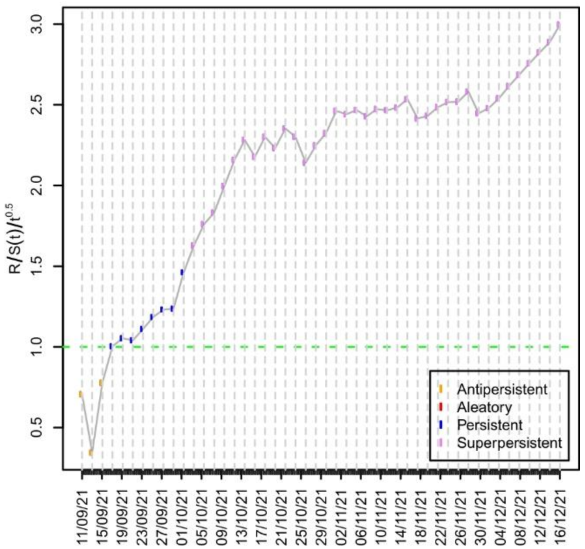

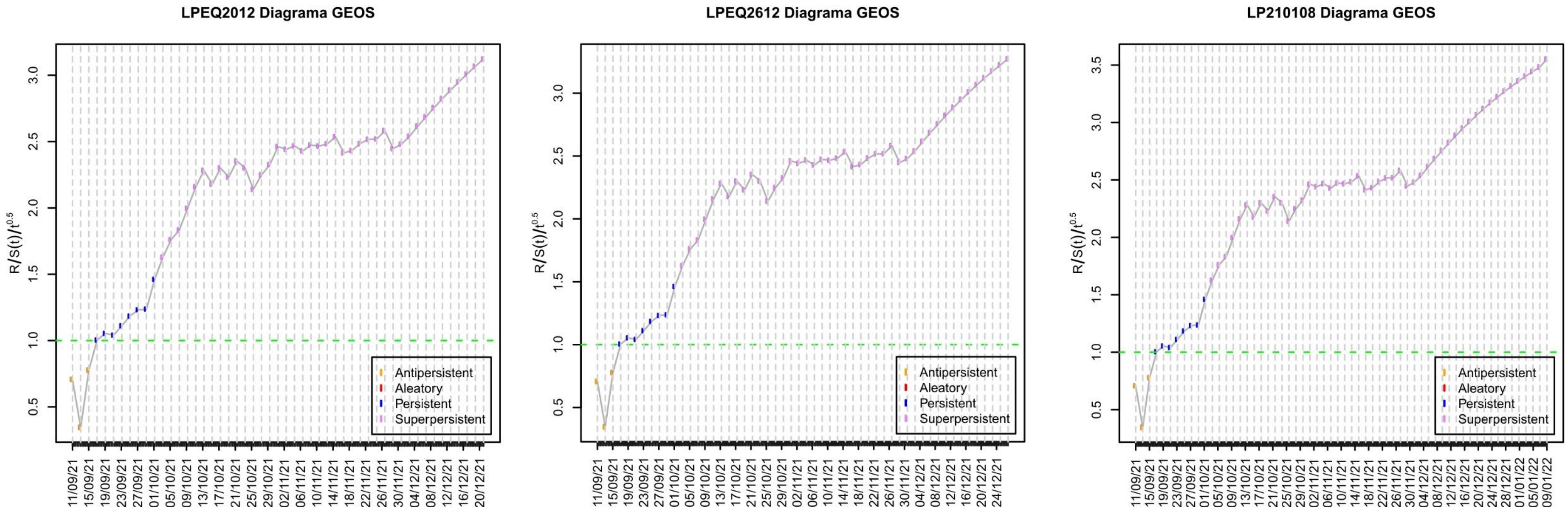


**Figure S2.** GEOS diagrams obtained during different days, from the R-code for the VT earthquakes during the volcanic Eruption of Cumbre Vieja, La Palma, 2021.
