# Supplementary material for: Anticipating volcanic eruptions using rescaled range analysis of volcano-tectonic seismicity
Source: Sci Rep. 2025 Dec 29;15:44803. doi: 10.1038/s41598-025-28566-6 (PMC12748848; doi:10.1038/s41598-025-28566-6)
Supplement: Supplementary file 3 — Supplementary Information 3. [file 41598_2025_28566_MOESM3_ESM.pdf]

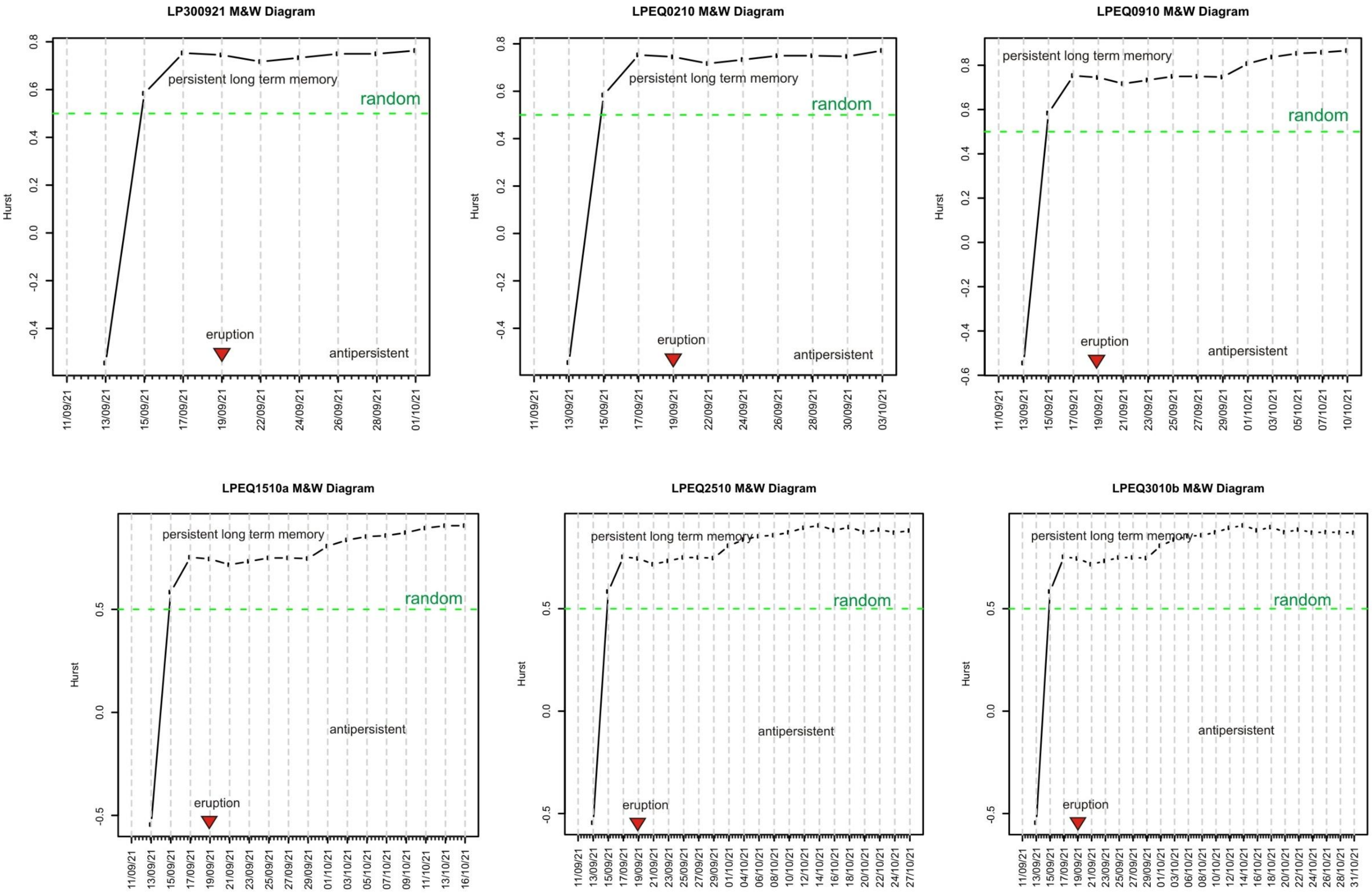
LP300921 M&W Diagram
LPEQ0210 M&W Diagram
LPEQ0910 M&W Diagram
LPEQ1510a M&W Diagram
LPEQ2510 M&W Diagram
LPEQ3010b M&W Diagram
Hurst
persistent long term memory
random
eruption
antipersistent

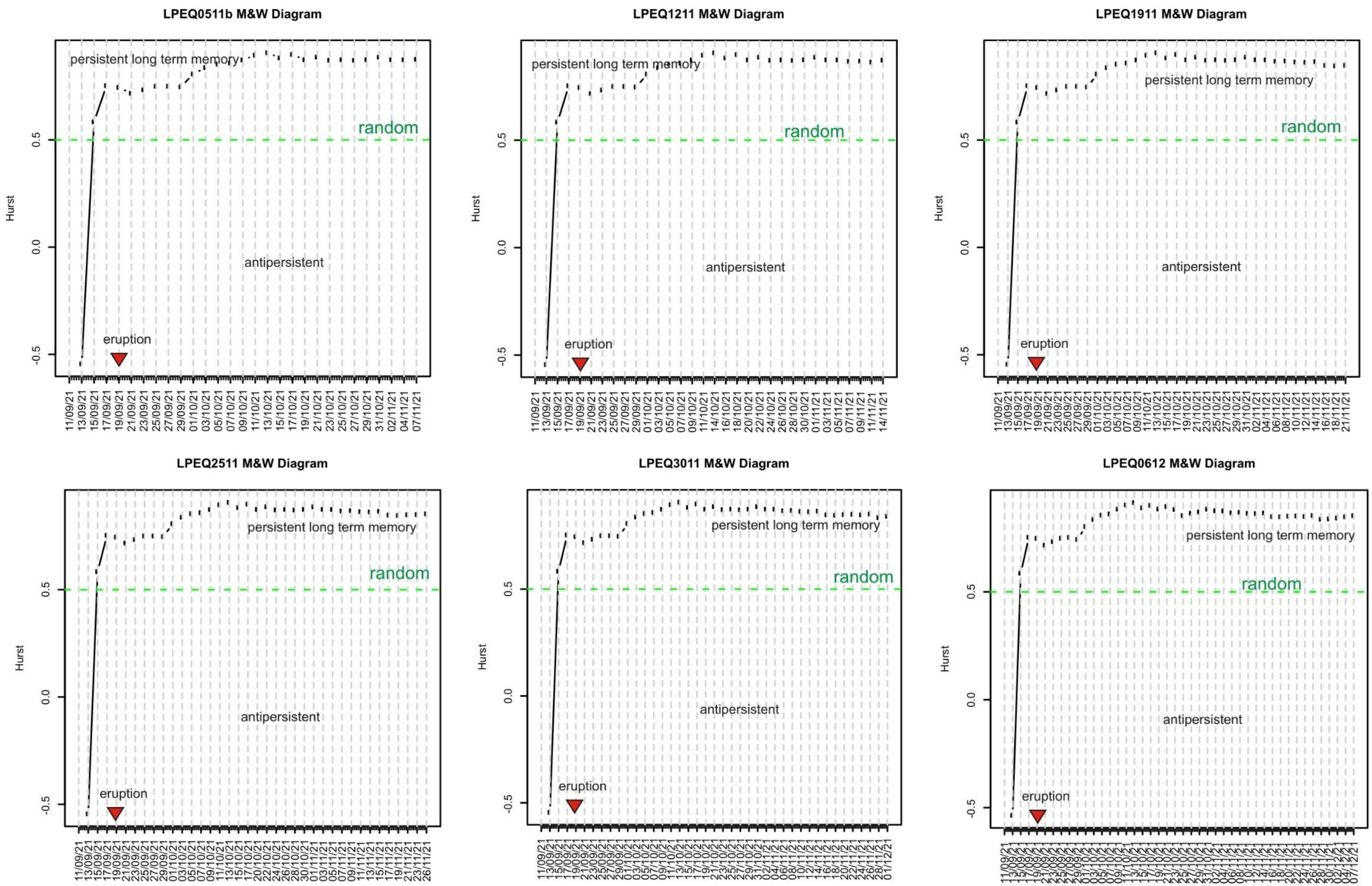
LPEQ0511b M&W Diagram
LPEQ1211 M&W Diagram
LPEQ1911 M&W Diagram
LPEQ2511 M&W Diagram
LPEQ3011 M&W Diagram
LPEQ0612 M&W Diagram
persistent long term memory
random
antipersistent
eruption
Hurst
0.5
0.0
-0.5

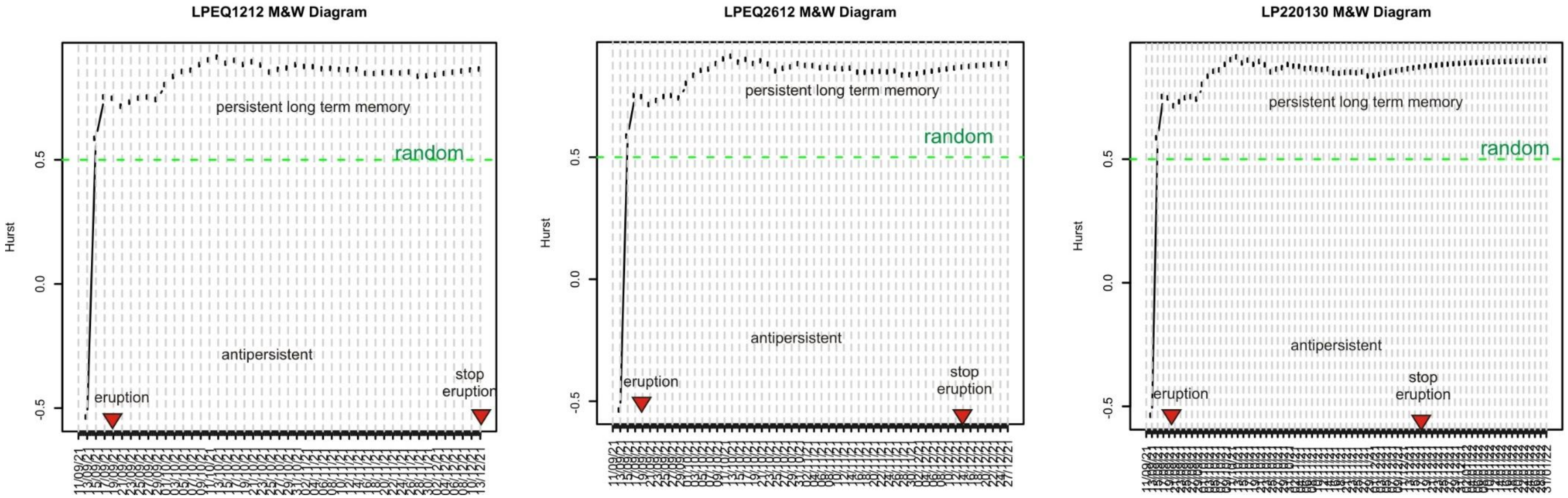

Figure S3. Mandelbrot and Wallis (M&W) diagrams of the Hurst exponent obtained from the R-code for the VT earthquakes during the volcanic Eruption of Cumbre Vieja, La Palma, 2021.
