# Supplementary figures and images for: Anticipating volcanic eruptions using rescaled range analysis of volcano-tectonic seismicity
Source: Sci Rep. 2025 Dec 29;15:44803. doi: 10.1038/s41598-025-28566-6 (PMC12748848; doi:10.1038/s41598-025-28566-6)

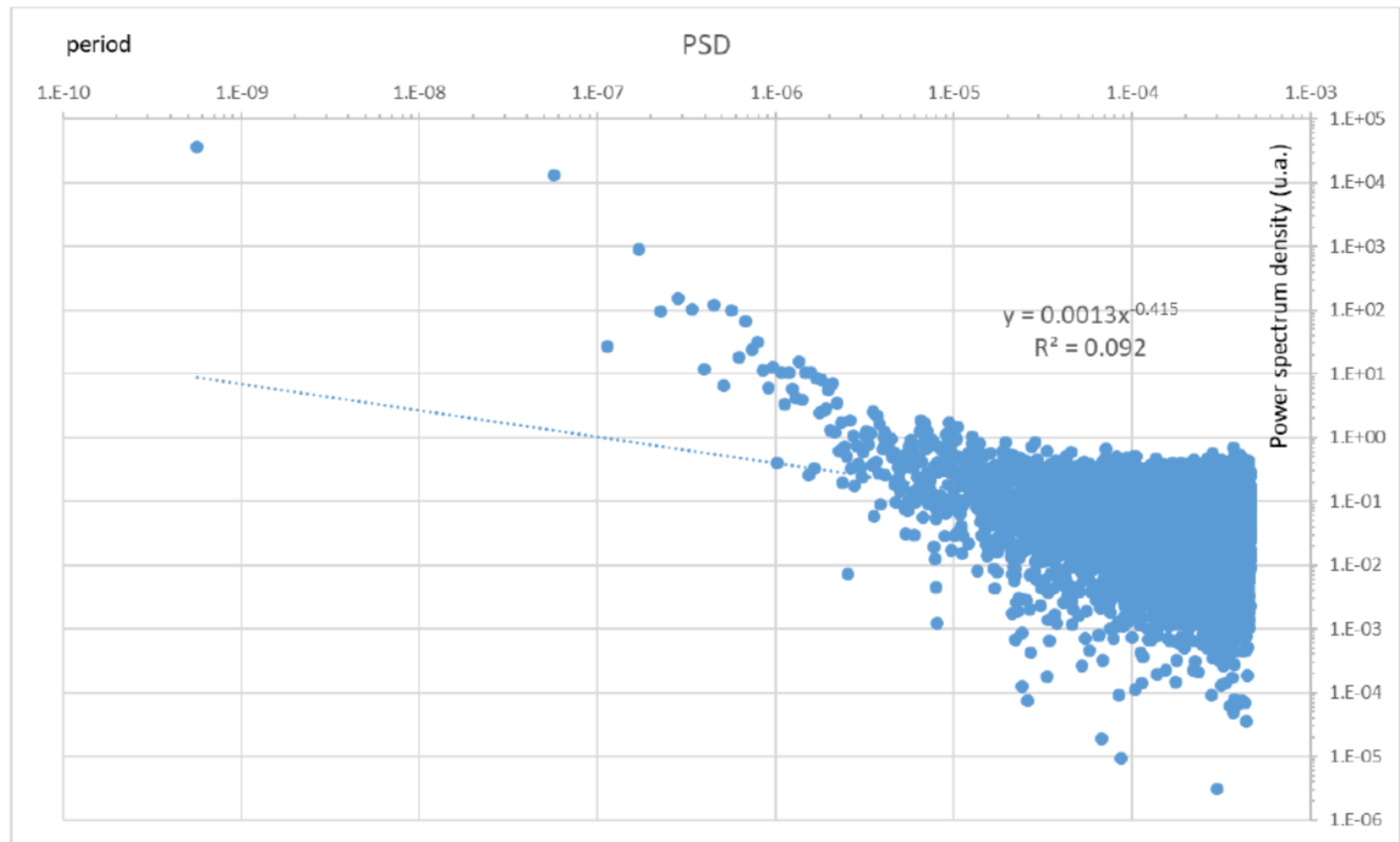

**Figure S4.** Power spectral density function of the VT earthquakes recorded by the IGN<sup>32</sup>.

Supplement: Supplementary file 4 — Supplementary Information 4. [file 41598_2025_28566_MOESM4_ESM.pdf]
